# Collective photon emission and ferroelectric exciton ordering near Mott insulating state in WSe_2_/WS_2_ heterobilayers

**DOI:** 10.1038/s41563-025-02476-4

**Published:** 2026-01-29

**Authors:** Luka Matej Devenica, Zach Hadjri, Jan Kumlin, Daniel Suárez-Forero, Runtong Li, Klevis Domi, Bosai Lyu, Weijie Li, Ludivine Fausten, Valeria Vento, Nicolas Ubrig, Song Liu, James Hone, Kenji Watanabe, Takashi Taniguchi, Thomas Pohl, Ajit Srivastava

**Affiliations:** 1 Department of Quantum Matter Physics, University of Geneva, Geneva, Geneva Switzerland; 2 Department of Physics, Emory University, Atlanta, USA; 3 Institute for Theoretical Physics, TU Wien, Vienna, Austria; 4 Department of Mechanical Engineering, Columbia University, New York, NY USA; 5 Institute of Microelectronics, Chinese Academy of Sciences, Beijing, China; 6 Research Center for Electronic and Optical Materials, National Institute for Materials Science, Tsukuba, Japan; 7 International Center for Materials Nanoarchitectonics, National Institute for Materials Science, Tsukuba, Japan

**Keywords:** Two-dimensional materials, Electronic properties and materials

## Abstract

Spontaneous symmetry breaking, driven by competing interactions and quantum fluctuations, is fundamental to understanding ordered electronic phases. Although electrically neutral, optical excitations like excitons can interact through their dipole moment, raising the possibility of optically active ordered phases. The effects of spontaneous ordering on optical properties remains underexplored. The excitonic Mott insulating state recently observed in semiconducting moiré crystals may help clarify this question. Here we present evidence for an in-plane ferroelectric phase of dipolar moiré excitons driven by strong exciton–exciton interactions. We reveal a speed-up of photon emission at late times and low densities in excitonic decay. This counterintuitive behaviour is attributed to collective radiance, linked to the transition between disordered and symmetry-broken ferroelectric phases of moiré excitons. Our findings provide evidence for strong dipolar intersite interactions in moiré lattices, demonstrate collective photon emission as a probe for moiré quantum materials and a path for exploring cooperative optical phenomena in strongly correlated systems.

## Abstract (web summary)

Ferroelectric ordering of moiré excitons and collective photon emission emerge in WSe_2_/WS_2_ heterobilayers near the Mott state driven by strong dipolar interactions and a transition to symmetry-broken ferroelectric phases.

## Main

Moiré crystals of stacked semiconducting transition metal dichalcogenides are being vigorously investigated for strongly correlated and topological electronic phases^1–9^. The electronic potential at moiré length scales also affects excitons and the moiré excitonic wavefunction has support primarily at moiré sites^10–12^. A natural question is whether, in addition to electronic phases, strongly correlated phases of lattice excitons exist in moiré quantum materials. At unit filling, on-site interactions between excitons in a lattice result in a correlated excitonic state that resembles a Mott insulator. Recent observations of this phase have opened the field for studying the Bose–Hubbard physics of excitons^13–18^.

In type-II semiconducting heterostructures, electrons and holes localize in separate layers, leading to long-lived, spatially indirect interlayer excitons (IXs). IXs feature an out-of-plane static dipole moment, making good candidates for realizing phases arising from finite-range interactions^19–23^. The additional moiré potential gives a richer internal structure to moiré excitons, leading to different excitonic properties and interactions. Moiré unit cells, which may be thought of as ‘moiré atoms’, can contain multiple minima for electrons and holes, allowing for different ‘moiré orbitals’. Electrons and holes in different moiré orbitals can, thus, have in-plane separation that greatly exceeds the out-of-plane layer separation, resulting in a large static dipole moment and, consequently, strong exciton–exciton interactions. Excitons are naturally dissipative towards photon emission, which we use as a probe of ordered phases and to explore possible collective emission through exciton–exciton interactions^24–26^.

We provide evidence for the in-plane ferroelectric ordering of static dipoles of moiré excitons in an H-stacked WSe_2_/WS_2_ heterobilayer close to the excitonic Mott state. Although the on-site hole interaction converts in-plane quadrupolar excitons into dipolar excitons above a certain filling, their finite-range dipole–dipole interactions close to unit filling align the dipoles in energetically favourable head-to-tail configurations. Given the moiré orbitals discussed above, the excitonic quantum state has three-fold degeneracy of the in-plane dipole orientation, corresponding to a discrete rotational symmetry. The full hybridization of these states implies a vanishing net static dipole moment and results in threefold enhancement of the excitonic photoemission rate. In contrast to such quadrupolar excitons, the symmetry-broken state with a maximal in-plane dipole features a three times longer radiative lifetime. Unlike atomic systems, the hybridization that causes collective light emission is not mediated by electromagnetic vacuum, but arises from tunnel coupling of the hole across the three configurations.

## Excitonic Mott insulator in H-stacked WSe_2_/WS_2_

**Fig. 1:**
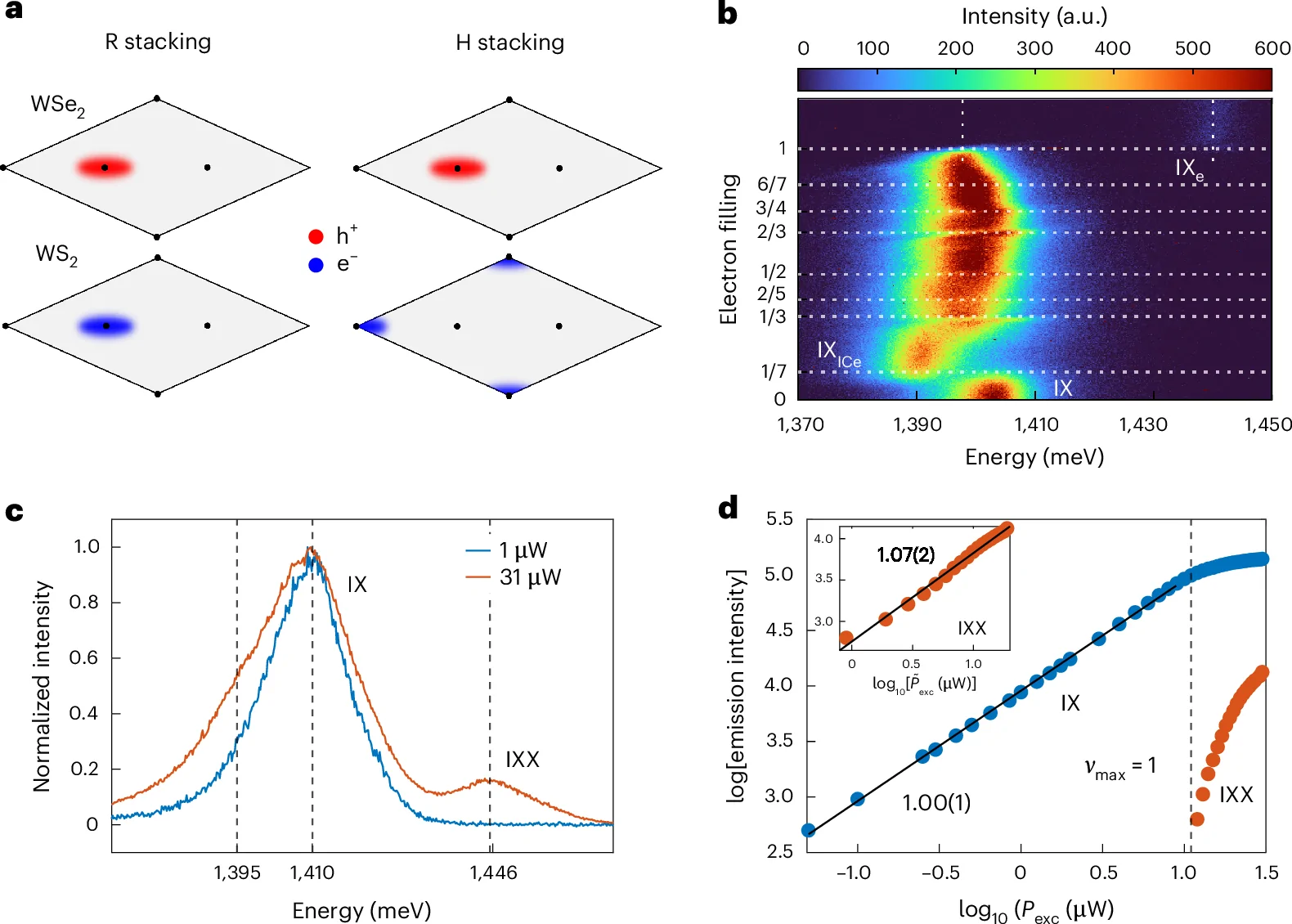
IXs in a WSe_2_/WS_2_ bilayer and their Mott insulating state. **a**, Diagram of electron and hole densities in an R- and H-stacked WSe_2_/WS_2_ heterobilayer moiré unit cell. The red (blue) areas indicate regions of high hole (electron) density. The black line indicates the edge of a moiré unit cell, and the vertices indicate the high-symmetry registry points. Unlike that in R stacking, an exciton in H stacking features a static in-plane quadrupolar moment. **b**, Doping scan of the PL emission of an H-stacked region, using 10 nW of 1-MHz 640-nm pulsed laser excitation. The dashed lines indicate generalized Wigner crystals at fractional fillings. **c**, PL emission of an H-stacked region at high and low excitation power, using a 1-MHz 640-nm pulsed laser. The difference in IXX to IX energy is *U*_IXX_ = 36 meV. At higher power, a red shoulder at 1,395 meV becomes prominent. **d**, Log–log plot of the total intensity of IX and IXX peaks as a function of excitation power. The intensity of IX increases linearly (slope, 1.00 ± 0.01) until the power at which IXX becomes measurable, when IX starts to saturate. The inset shows the IXX intensity against power, with an offset to the IX saturation power ${\widetilde{P}}_{exc}={P}_{exc}-{P}_{exc}({\nu }_{\max }=1)$, which shows that the IXX intensity also increases linearly (slope, 1.07 ± 0.02)) when the power is above the saturation value. Source data

Figure 1a shows the relative positions of electrons and holes in the moiré unit cells of a lattice-mismatched WSe_2_/WS_2_ heterostructure, as determined by density functional theory calculations in previous works^16,18^. In the R-stacked (0° stacking angle) case, the potential minima for the hole and electron have no lateral displacement; therefore, the exciton has a static out-of-plane dipole, but no in-plane dipole or higher-order moment. By contrast, the H-stacked (60° stacking angle) case features laterally displaced potential minima of the electron and hole. For a given position of the hole (electron) of the exciton, the electron (hole) is free to delocalize over the three equivalent neighbouring sites, resulting in an in-plane quadrupolar moment (Fig. 1a). In this work, we focus on H stacking to explore the resulting in-plane quadrupolar and dipolar nature of the moiré excitons.

To confirm the presence of a moiré superlattice in our sample, we studied photon emission as a function of carrier density. Figure 1b shows the photoluminescence (PL) emission from IX in the H-stacked region under electron doping. Our dual-gated sample (Supplementary Fig. 1) permits the independent control of doping and out-of-plane displacement field. Consistent with previous observations^14–18^, we measure a blueshifted peak in PL (IX_e_) at unit electron filling, which arises from the on-site exciton–electron repulsion (*U*_Xe_ ≃ 42 meV). We observe a characteristic modulation of IX PL from 1/3 to unit filling, consistent with the formation of generalized Wigner crystals of electrons at fractional fillings^5,6,27^. An exception to this pattern occurs at 1/7 filling, where we observe a sudden and sizeable (~13 meV) redshift in emission. As in previous work^16^, we interpret this as the formation of an intercell exciton complex between an exciton and an electron in neighbouring moiré sites. The quadrupolar IX becomes polarized by attractive interaction with a neighbouring electron, and the 1/7 electron Wigner crystal guarantees that each exciton will have at least one neighbouring IX (Supplementary Fig. 2). Indeed, at 1/7 electron doping, all IXs are redshifted. Therefore, we label the state at 1/7 filling as the intercell IX–electron complex (IX_ICe_). Importantly, this redshift was not seen in previous studies on R stacking, where electrons and holes have no in-plane displacement. This difference provides strong evidence for the in-plane quadrupolar nature of moiré excitons in H-stacked bilayers^16,18^. Measurement of the IX *g*-factor of 10.5 ± 0.5 (Supplementary Fig. 3) confirms our assignment of the H-stacked region^6,28^.

Having established the presence of a moiré lattice, we turn to photon emission. Using pulsed laser excitation, we can measure the dependence of the PL spectrum on excitation power (Methods). As shown in Fig. 1c, at a certain power threshold, another blueshifted peak appears. In accordance with recent works^14,15,17,18^, we attribute this peak (IXX) to two excitons sharing a single moiré site. We further confirm this by observing IXX’s lack of circular polarization^16^ (Supplementary Fig. 4). By comparing the spectra at low and high excitation powers, we deduce an on-site repulsion energy of ~36 meV, which makes the state at exactly one exciton per moiré site filling an excitonic Mott insulator. The total emission intensities of IX and IXX further confirm the Mott state (Fig. 1d). The IX intensity increases linearly with excitation power until we observe the additional IXX peak, and the IX intensity begins to saturate. We label the power at which we first observe IXX emission as ${\nu }_{\max }=1$ (Supplementary Note 1). Beyond ${\nu }_{\max }=1$, the IXX peak grows linearly (Fig. 1d, inset). This means that IXX appears only when all the available nearby IX sites are filled (Supplementary Figs. 5 and 6). The linear power dependence of the IX intensity below ${\nu }_{\max }=1$ permits a direct conversion between excitation power and exciton filling ${\nu }_{\max }$ with a high degree of accuracy (Supplementary Note 1).

## IX–IX redshift in H-stacked samples

**Fig. 2:**
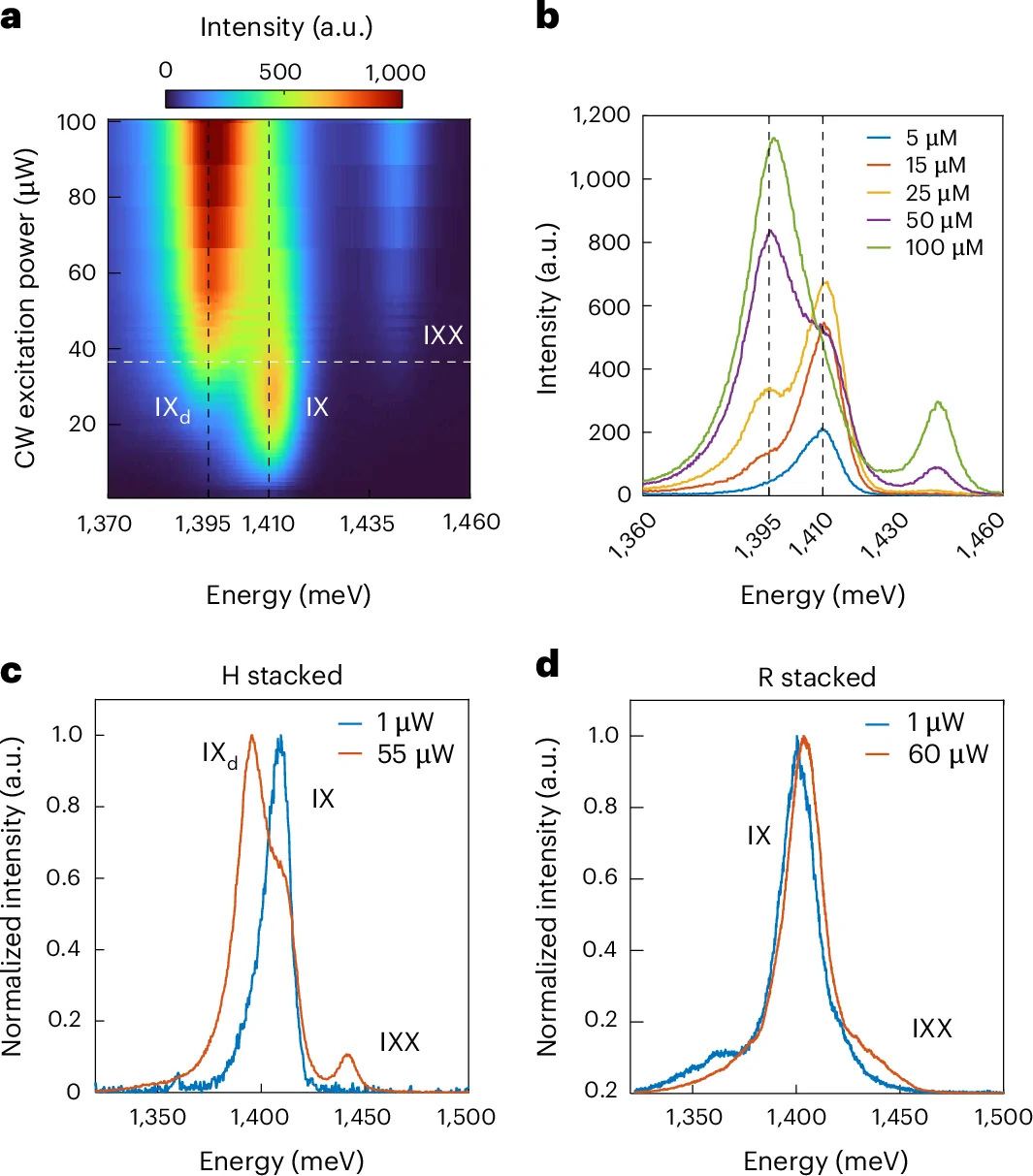
Redshifted IX emission at high excitonic density. **a**, IX PL as a function of excitation (640-nm CW laser) power at charge neutrality. The horizontal dashed line indicates the power at which IXX appears. **b**, Select spectra from **a** showing an IX_d_ redshift of 15 meV. The IX_d_ peak appears before IXX and increases with power, at the expense of the IX peak. **c**, Normalized IX PL spectra at low and high (640-nm CW laser) power in the H-stacked region of the sample. **d**, Normalized IX PL spectra at low and high (640-nm CW laser) power in the R-stacked region of the sample. Source data

To explore the effects of exciton–exciton interactions in the moiré lattice, we study the effect of exciton density on emission. We measured the power dependence of PL spectrum under continuous-wave (CW) driving, which establishes a steady-state exciton density. As shown in Fig. 2a,b, we observe a redshifted peak emerge at an excitation power below the critical power for creating IXX. With increasing pump power, this redshifted exciton signal, which we denote as IX_d_, rapidly exceeds the low-density IX peak and eventually dominates the exciton emission at high densities. Importantly, we do not observe the redshifted IX_d_ peak in R-stacked regions of the sample under identical CW excitation (Fig. 2c,d). The opposite slope of the Stark shift with the displacement field in the R- and H-stacked regions comes from our sample geometry (Supplementary Fig. 1), and confirms our assignment of the measured regions (Supplementary Fig. 7). This indicates that the redshifted IX_d_ peak originates from excitonic in-plane dipoles, which are only present in the H-stacked case. The coexistence of IX and IX_d_, together with the absence of a gradual energy shift with increasing powers, rules out power-dependent sample heating as a cause for the emergence of IX_d_. Below, we present further evidence that this surprising density dependence of the exciton emission arises from exciton–exciton interactions, due to their in-plane dipoles in H-stacked bilayers.

## Density-dependent IX dynamics

**Fig. 3:**
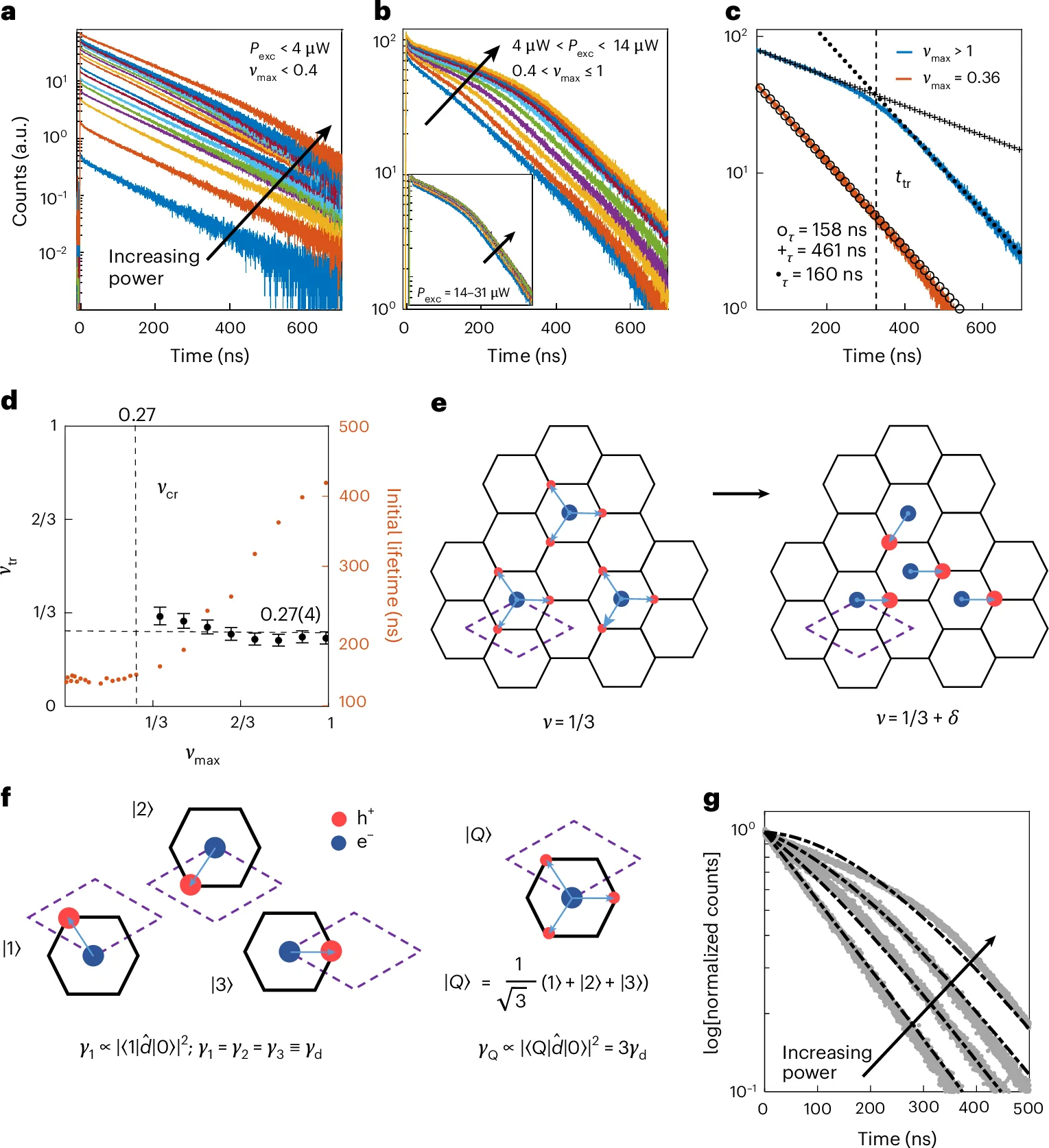
Temporally and spectrally resolved IX emission in the H-stacked region. **a**, IX emission decay for the low-excitation-power regime (${\nu }_{\max } <$ 0.4). The decay is exponential and the lifetime is constant over the entire power range. The power indicated in **a**–**c** is the average excitation power, using a 640-nm, 1-MHz pulsed laser. The collected emission in **a**–**c** is filtered to only include IX and IX_d_ (Methods). **b**, IX emission decay for the intermediate-excitation-power regime ((0.4 $< {\nu }_{\max } <$ 1)). The decay becomes initially slower and then returns to the low-density decay rate. The inset shows the IX emission decay for the high-excitation-power regime (${\nu }_{\max } >$ 1). The slowing effect has saturated beyond the excitonic Mott state. Axis scaling and limits on the inset are the same as in main panel. **c**, IX emission decay at high (blue) and low (orange) excitation densities. The fits show slow and fast decay rates occurring in the high- and low-density cases, respectively. The dashed line indicates the time of transition ${t}_{tr}$ from the slow- to the fast-decay regime, determined by the intersection of the two fits. At the transition point ${t}_{tr}$, the exciton density is ${\nu }_{tr}$. **d**, Black data (left axis) show the transition fraction ${\nu }_{tr}$ as a function of the initial density ${\nu }_{\max }$, determined by the method shown in **c**. The dashed line indicates the mean observed ${\nu }_{tr}$ of 0.27 (4). The error bars are derived from the range of possible intersection points of the two fits, using the uncertainties of the fitted parameters. Note that ${\nu }_{tr}$ is expressed in units of ${\nu }_{\max }$. Orange data (right axis) show the initial (first 10–100 ns) lifetime of emission as a function of the initial density ${\nu }_{\max }$. The power at which ${\nu }_{\max }$= *ν*_cr_ is indicated by the dashed line, and coincides with the point at which a slower initial lifetime is first measured. **e**, Schematic of quadrupolar excitons at 1/3 filling. Moiré unit cell is indicated using purple dashed lines, and the hexagonal full lines indicate the effective electronic lattice, with each electron site at the centre of a hexagon. At 1/3 filling, assuming a homogeneous exciton distribution, every empty site is adjacent to at least one quadrupolar exciton. When an additional exciton is added, it necessarily resides next to three neighbours and the excitons may acquire a non-zero in-plane dipole moment due to carrier repulsion. **f**, Diagram of decay rates of excitons with static in-plane dipolar and quadrupolar moments. Excitons with a static dipolar moment form a triply degenerate basis, due to the triangular symmetry of the H-stacked potential. The oscillating dipole moment of the static quadrupolar state (*γ*_Q_) is an equal superposition of the oscillating dipole moments of the static dipolar states (*γ*_d_), and is expected to have a three times faster collective decay rate (*γ*_Q_ = 3*γ*_d_). The purple dashed lines indicate moiré lattice edges. **g**, Model simulations (dashed line) of power-dependent lifetime data (grey; see Supplementary Note 4). Source data

To this end, we measured the exciton decay dynamics after pulsed excitation (Methods). Figure 3a,b shows the time evolution of the total PL signal from combined IX and IX_d_ peaks for different initial lattice filling fractions ${\nu }_{\max }$, controlled by the power *P*_exc_ of the excitation pulse. Up to an initial excitonic filling of ${\nu }_{\max }\approx 0.36$, we observe simple exponential decay with a lifetime of 160 ± 10 ns. As the filling increases beyond this value, the initial decay dynamics slow down steadily (Fig. 3b). The R-stacked region of the sample and an additional R-stacked sample do not exhibit this slowdown (Supplementary Fig. 8). The initial decay time saturates at almost three times greater (460 ± 10 ns) near the excitonic Mott state (${\nu }_{\max }=1$). We rule out unintentional photodoping as a reason for this slowdown (Supplementary Note 3 and Supplementary Fig. 9). At longer times, the excitonic decay speeds up to the same decay time found at lower initial densities. Increasing the excitation power past unit filling has no effect on the decay dynamics (Fig. 3b, inset). This indicates that excitons in doubly occupied sites (IXX) generated at high excitation powers undergo rapid decay, leaving behind a near-unit-filled lattice. This process explains the small non-monotonic ‘burst’ in the first 30 ns of the high-power data (Supplementary Note 2 and Supplementary Fig. 6c). These decay dynamics are surprising, as an increased exciton density often accelerates decay because of non-radiative processes^29,30^ and fast processes tend to rapidly deplete, leading to fast decay followed by slow decay^31–33^.

For a more detailed analysis, we fit the two regimes in Fig. 3b with exponentials and define the transition time ${t}_{tr}$ at which the two curves intersect (Fig. 3c). By integrating the decay curve before and after this point, we can determine the filling fraction of excitons remaining at the transition (${\nu }_{tr}$). Figure 3d shows ${\nu }_{tr}$ as a function of initial filling ${\nu }_{\max }$, and demonstrates that the exciton decay rate changes at a constant filling fraction of ~0.27 ± 0.04. In Fig. 3d, we also show the initial decay lifetime versus ${\nu }_{\max }$. As discussed above, the initial decay time remains independent of filling until a critical value *ν*_cr_, followed by a gradual increase to a threefold value at ${\nu }_{\max }=1$. Crucially, this critical value (${\nu }_{cr}\approx {\nu }_{tr}$) coincides with the filling fraction at which the slow-to-fast transition occurs. This provides strong evidence that exciton density effects are causing a slowdown in photon emission above a lattice filling of ~1/3.

To understand this puzzling slow-to-fast decay transition, we focus on the filling fraction (${\nu }_{tr}\approx 1/3$), which marks the transition between slower initial decay and faster asymptotic decay. As depicted in Fig. 3e, for fillings above ${\nu }_{\max }$= 1/3, there would be a guaranteed carrier–carrier overlap if all excitons remained quadrupolar. For two adjacent excitons, the strong on-site Coulomb repulsion between holes (~50 meV)^6,34^ greatly exceeds the energy of hybridizing the three states of each exciton. This constraint on hole delocalization locally breaks the three-fold rotational symmetry such that excitons acquire an in-plane dipole moment (Fig. 3e). Therefore, for ${\nu }_{\max } > 1/3$, the ratio of dipolar-to-quadrupolar excitons increases. Indeed, this critical density is close to the observed values of ${\nu }_{tr}$ and *ν*_cr_ for the transition between slow and fast decay.

Let us consider the effect of such a polarization of excitons on their radiative decay rate. For a given electron position in the lattice, an isolated moiré exciton (*ν*_X_ < 1/3) can form in three degenerate states, corresponding to different orientations of the static excitonic dipole and which we denote as  ∣1〉,  ∣2〉 and  ∣3〉 (Fig. 3f). The rate of photon emission *γ*_d_ from each of these polarized dipolar states is proportional to the square of the transition dipole moment ($\widehat{d}$):

1 $${\gamma }_{{\rm{d}}}\propto {| \langle i| \widehat{d}| 0\rangle | }^{2},i=1,2,3,$$

where  ∣0〉 denotes the vacuum state at a given site. Owing to their three-fold rotational symmetry, we expect isolated moiré excitons to form a hybridized symmetric superposition state $| Q\rangle =\frac{1}{\sqrt{3}}(| 1\rangle +| 2\rangle +| 3\rangle )$, minimizing kinetic energy. The deep subwavelength nature of the moiré lattice ensures that the spatial variation of the electromagnetic field across a single moiré lattice site can be neglected. In fact, this quadrupolar exciton can be considered as a few-body superradiant state, which—due to its deep subwavelength spatial extent—features a collectively enhanced photon emission rate:

2 $${\gamma }_{{\rm{Q}}}\propto {| \langle Q| \widehat{d}| 0\rangle | }^{2}={\left|\frac{\langle 1| +\langle 2| +\langle 3| }{\sqrt{3}}\widehat{d}| 0\rangle \right|}^{2}=3{\gamma }_{{\rm{d}}}.$$

Note that the transition dipole moment ($\widehat{d}$) is unrelated to the static dipole, such that the polarization of emitted photons does not depend on the orientational state (∣i⟩) of the static dipole. Hence, we conclude that the fully hybridized quadrupolar exciton should decay three times faster than a polarized dipolar exciton.

We performed microscopic simulations of the correlated decay dynamics using a Monte Carlo approach (Supplementary Note 4). For a given spatial configuration of excitons in the lattice, we perform a Monte Carlo sampling of their dipoles, including the orientational constraint from local hole repulsion. This yields corresponding decay rates that propagate the system via Monte Carlo sampling of stochastic quantum jumps. Offering full information about the time-evolving many-body state of the lattice, this approach yields the dynamic and spectral properties of photon emission. Figure 3g compares the simulation results and measured signal for different initial lattice fillings (Supplementary Note 5 and Supplementary Fig. 10). We find fair agreement between the experiment and simulations, considering the simplicity of the theoretical description. Importantly, the theory reproduces the observed crossover from the initially slow decay to a collectively enhanced decay at long times and high initial lattice filling (*ν*_X_ ≳ 1/3). At higher densities, the simulations show non-exponential initial decay without the sharp transition between two exponential-decay regimes seen in experiments. Given the simplicity of the proposed model to analyse the basic physical mechanisms behind observed decay dynamics, we anticipate improved quantitative agreement on extending the present theory by accounting for the additional orientational constraints arising from finite-range dipole–dipole interactions between nearby excitons.

## Density-dependent IX spectrum

**Fig. 4:**
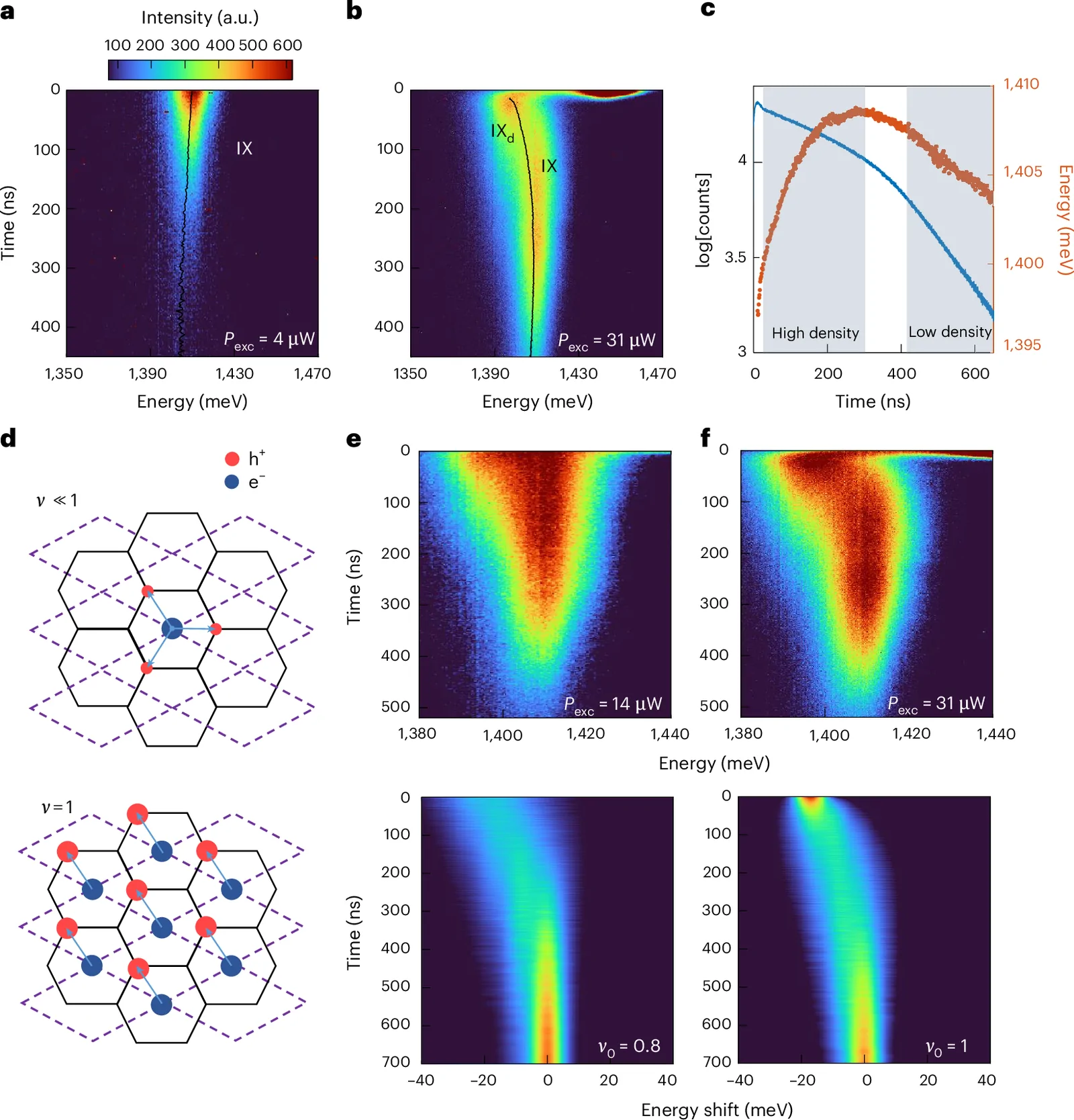
Experimental study and modelling of the density-dependent IX lifetime and energy dynamics in the H-stacked region. **a**, Time-resolved IX spectrum at ${\nu }_{\max }=$ 0.4, using a 640-nm pulsed laser. The black line indicates the fitted peak centre. **b**, Time-resolved IX spectrum at ${\nu }_{\max } >$ 1, using a 640-nm pulsed laser. The black line indicates the fitted peak centre. The colour bar is the same as that in **a**. **c**, Decay profile and fitted peak centre position of the data in **b**. The emission centre is initially redshifted and blueshifts during the period in which the decay is slow. **d**, Diagram of transition from quadrupolar, low-density exciton to a ferroelectric phase at unit filling. **e**, Comparison of the measured (top) time-dependent PL at *P*_exc_ = 14 µW to simulation (bottom). The simulation panels show intensity-normalized spectra to emphasize the emission energy. Simulation details are given in Supplementary Note 3. The colour bar is the same as that in **a**. **f**, Comparison of measured (top) time-dependent PL at *P*_exc_ = 31 µW to simulation (bottom). The colour bar is the same as that in **a**. Source data

Our theoretical picture suggests close correlation between the two-stage decay dynamics and nonlinear spectral response of moiré excitons in our sample. We explore this question further by measuring the time-resolved PL spectra (TRPLS) with an electronic streak-camera technique (Methods and Supplementary Fig. 11) to compare the PL emission energies from the IX and IX_d_ peaks in the two lifetime regimes. Spectrally binning the data in the same range used for the lifetime measurements confirms that the observed TRPLS decay is consistent with lifetime measurements discussed above (Supplementary Fig. 12). Figure 4a shows the TRPLS at low excitation power, where we observe exponential decay with a single rate of ~3*γ*_d_. However, at high excitation powers (Fig. 4b), the emission starts as redshifted IX_d_, whose energy shift of 15 meV coincides with that observed in the CW experiments (Fig. 2a,b). As excitons decay and their density decreases, the emission energy returns to that of the IX peak, seen at low initial densities (Fig. 4a). Figure 4c shows the dynamics of the average emission energy together with the total PL signal. The measurement demonstrates that the energy transitions gradually from that of the dipolar IX_d_ exciton and reaches its maximum value corresponding to the quadrupolar IX during the slow initial regime of photon emission. The observed time evolution is consistent with an initially ordered ferroelectric phase at high densities with a lowered energy due to in-plane interactions between aligned dipoles (Fig. 4d), and whose energy rises as excitons decay until a filling of ~1/3 in which the system is primarily composed of isolated quadrupolar IX (Fig. 4d). After transitioning to IX, a subsequent smaller 3-meV redshift is observed, which also occurs in the low-power case (Fig. 4a–c), and most probably arises from decreasing out-of-plane dipolar repulsion with decreasing densities^15,18^. This conclusion is further supported by the fact that repulsive dipolar shifts are also observed for R stacking (Fig. 2d). We note that the static in-plane displacement of the electron and hole in the dipolar configuration has no effect on the transition dipole moment responsible for emission. Consequently, the ferroelectric ordering of excitons does not result in linearly polarized emission. To confirm our experimental results, we studied an additional H-stacked WSe_2_/WS_2_ bilayer. In agreement with the first sample, the additional sample exhibits a blueshifted IXX peak and redshifted IX_d_ at higher excitation powers (Supplementary Figs. 13 and 14). Additionally, slowdown in the initial decay dynamics is observed for $0.3\lesssim {\nu }_{\max }\lesssim 1$ (Supplementary Fig. 15), with corresponding *ν*_cr_ ≈ 0.24 (Supplementary Fig. 16). Moreover, TRPLS shows the redshifted IX_d_ present at early times, coinciding with the slower emission rate (Supplementary Fig. 17), thereby reproducing all the characteristic features of the first sample. However, the saturation of IX with the appearance of IXX, the slowdown of the decay dynamics, and the intensity and redshift of IX_d_ are not as pronounced as in the first sample. These quantitative differences could arise from twist-angle inhomogeneities or disorder-induced lowering of the three-fold degeneracy of the moiré potential minima across different samples.

For quantitative analysis, we can use our Monte Carlo simulations to calculate the expected energy of the TRPLS data. Having access to microscopic exciton configurations during the simulated decay dynamics, we determine the energy of emitted photons from the dipole–dipole interaction energy at every Monte Carlo step. Including a Gaussian linewidth of Δ*E* = 10 meV to account for typical broadening effects and averaging over many Monte Carlo runs, we obtain the time-evolving emission spectra (Fig. 4e,f). Indeed, the calculations capture the essential features of our TRPLS measurements. Both experiment and theory show the characteristic change in emission spectrum due to the formation of a ferroelectric phase of ordered dipoles at high densities. We find that an in-plane dipole moment corresponding to ~70% of the distance between the single-particle potential minima of the electron and hole reproduces the observed ~15-meV redshift in the fully polarized ferroelectric phase. Owing to electron–hole attraction, such a reduction in the excitonic electron–hole separation appears reasonable since the moiré potential depth is comparable with the exciton binding energy^35,36^.

Our observations reveal rich interplay between on-site Coulomb interactions, finite-range dipole–dipole interactions and collective photon coupling of excitons from in-plane excitonic dipole moments in moiré quantum materials. The physics of ferroelectricity in two-dimensional materials has attracted substantial interest, motivated by broad scientific and technological perspectives^37^. The demonstrated dynamical transition to ferroelectric ordering could be combined with in-plane electric fields to tune the spontaneous macroscopic polarization in optically active quantum materials. Our findings suggest new scientific venues—from studying emergent ordered phases in dipolar quantum matter in and out of equilibrium^38–41^ to exploring cooperative optics in ordered strongly interacting systems^24,42^. Extending the proposed model to account for finite-range dipole–dipole interactions may yield more accurate quantitative descriptions of observed photoemission dynamics and deepen our understanding of interaction effects on cooperative optical processes in quantum materials. Moreover, our results motivate future investigations into the non-equilibrium physics of the quantum rotor or the three-state Potts model^43,44^, the emission of non-classical light from strongly correlated states of quantum emitters^45,46^, or the probing and sensing of phases and phase transitions in quantum materials.

## Methods

### Device fabrication

We use a dry-transfer fabrication method with a polycarbonate (PC) stamp to fabricate the dual-gated, hexagonal boron nitride (hBN)-encapsulated transition metal dichalcogenide devices^47^. WSe_2_ and WS_2_ monolayers, few-layer graphene flakes for gates and contact, and hBN for encapsulation are mechanically exfoliated from bulk crystals onto 300-nm SiO_2_ on Si substrates. The thickness of the hBN flakes, estimated from optical contrast, ranges from 20 to 40 nm. First, we use a PC stamp to pick up the bottom hBN and the bottom few-layer graphene, which is then deposited onto a 300-nm SiO_2_/Si substrate with electron-beam-lithography-made electrodes (5-nm Cr/85-nm Au) at 170 °C. The substrate with bottom hBN and graphene is then annealed in a 5% H_2_/95% N_2_ environment at 350 °C for 3 h to remove the PC residue. The few-layer graphene top gate, top hBN flake, top WS_2_ monolayer, WSe_2_ monolayer, bottom WS_2_ monolayer and the few-layer-graphene contact are picked up in that order with a PC stamp and then placed onto the back gate at 170 °C. We use the tear-and-stack method^48^ to pick up half of a large WS_2_ flake as the top monolayer, align with the WSe_2_ layer within 1° uncertainty and pick up the other half of the WS_2_ flake with a 60° rotation relative to the top WS_2_, and offset the flakes laterally to create distinct R- and H-stacked regions, separated by a trilayer region (Supplementary Fig. 1).

### Optical measurements and electrostatic gating

PL spectroscopy and emission lifetime measurements were performed at two different base temperatures of 8 K (attoDRY 800) and 3.8 K (attoDRY 1000 and Bluefors BF-LD 4K), each featuring a custom-made confocal optical microscope. The data in the main text were collected in the attoDRY 800 system, with the exception of Fig. 1b, for which the data were collected in the Bluefors BF-LD 4K system. Lower temperatures led to longer observed absolute lifetimes, requiring a reduction in repetition rate to ensure that the signal completely decayed before the next pulse arrived. In each cryostat, we use a piezoelectric controller (attocube systems) to move the sample. The excitation laser used was PicoQuant LDH-D-C-640, with a centre wavelength of 640 nm and operated in the pulsed (up to 1 MHz) or CW mode. The pulsed experiments allow for a targeted initialization of a particular exciton population to the system, whereas the CW excitation establishes a steady-state excitonic density. In each setup, an achromatic objective (numerical aperture = 0.42 for attoDRY 800, numerical aperture = 0.63 for Bluefors BF-LD 4K and numerical aperture = 0.82 for attoDRY 1000) is used to focus the laser beams to a spot size of ~1 µm, which is then collected through the same objective and coupled into an optical fibre. The light was then directed either to a spectrograph or avalanche photodiode for emission lifetime measurements.

For spectrophotometry, light was outcoupled into a high-resolution spectrograph: a Princeton Instruments HR-500 (500-mm focal length) for the attoDRY 800 system, or a Princeton Instruments SP-2750i (750-mm focal length) for the Bluefors BF-LD 4K and attoDRY 1000 systems. Both spectrographs used a 300 grooves mm^−1^ grating (blazed at 750 nm). A charge-coupled device (Princeton Instruments PIXIS-400 charge-coupled device for attoDRY 800 and PyLoN charge-coupled device for Bluefors and attoDRY 1000) was used as the detector. The excitation and emission arms also featured a liquid crystal retarder each (Thorlabs LCC1513-B), which enabled us to excite the sample (and collect the emission of light) with well-defined linear or circular polarization.

For emission lifetime measurements, the light was outcoupled to an avalanche photodiode (PerkinElmer SPCM-AQR-16-FC), which was plugged into a photon counter (PicoQuant PicoHarp 300). The sample emission was filtered using a combination of 780-nm low-pass and 900-nm (full-width at half-maximum, 20 nm) band-pass filters to simultaneously collect IX and IX_d_ and exclude IXX.

For the TRPLS measurements, we used a fibre-coupled acousto-optic modulator (AOM; AeroDIODE 850-AOM-2) in-line with the fibre collection, which modulated the collection time window, after which the same setup as described in the previous paragraph was used to collect the spectra (Supplementary Fig. 11). The AOM on time was controlled by a tunable-width digital delay/pulse generator (SRS DG535), whose other channel also served as the trigger for the pulsed laser, ensuring synchronization across excitation and collection. The AOM on time was set to 10 ns by sending 10-ns-wide pulses from SRS DG535 and operating the AOM controller in the external-trigger mode. The delay of the AOM on-time pulse was then scanned, giving binned PL measurements over 10-ns intervals at arbitrary delays.

Through two source meters (Keithley 2400), we apply independent voltages to the graphene top gate and graphene bottom gate to tune the charge density or apply an electric field to the sample, whereas the contact to the sample is grounded to a ground common to the source meters.

### Emission lifetime and energy simulation

The emission lifetime and emission spectra simulations were performed using a Monte Carlo sampling and quantum jump approach on a triangular lattice with 30 × 30 sites under periodic boundary conditions (Supplementary Note 4). Excitons were treated as dipolar particles occupying distinct lattice sites, initialized in one of three possible hole configurations. For filling fractions below *ν* = 1/3, we avoid the occupation of nearest-neighbour sites due to dipolar repulsion and the fact that there is no slowdown in the decay measured below these filling fractions. Emission rates were determined by local exciton densities, with recombination probabilities calculated dynamically. For example, excitons with no occupied neighbouring sites decayed with a rate of *γ*_Q_ = 6.3 MHz (lifetime *τ*_Q_ = 160 ns), whereas those with fully occupied neighbouring sites had a reduced rate of *γ*_d_ = *γ*_Q_/3 = 2.1 MHz (lifetime *τ*_d_ = 480 ns), matching experimentally obtained high- and low-density lifetimes. Intermediate rates were assigned proportionally based on available hole states. The evolution followed a Lindblad master equation with dissipative recombination dynamics simulated through stochastic quantum jumps. Emission spectra were obtained by tracking energy shifts during the exciton decay, incorporating only nearest-neighbour Coulomb interactions. Quantitative agreement with the experimental data is obtained by reducing the in-plane distance of electrons and holes by a factor of 0.7 compared with their single-particle potential minima.

## Online content

Any methods, additional references, Nature Portfolio reporting summaries, source data, extended data, supplementary information, acknowledgements, peer review information; details of author contributions and competing interests; and statements of data and code availability are available at https://doi.org/10.1038/s41563-025-02476-4.

## Supplementary information

Supplementary Information Supplementary Figs. 1–17 and Notes 1–5.

## Source data

Source Data Fig. 1 Statistical source data.

Source Data Fig. 2 Statistical source data.

Source Data Fig. 3 Statistical source data.

Source Data Fig. 4 Statistical source data.

## Data Availability

All the data that support the findings of this study are reported in the Article and its Supplementary Information. Source data are provided with this paper.
