# Supplementary material for: Collective photon emission and ferroelectric exciton ordering near Mott insulating state in WSe2/WS2 heterobilayers
Source: Nat Mater. 2026 Jan 29;25(5):755–61. doi: 10.1038/s41563-025-02476-4 (PMC13649553; doi:10.1038/s41563-025-02476-4)
Supplement: Supplementary file 1 [file 41563_2025_2476_MOESM1_ESM.pdf]

nature materials

Supplementary information https://doi.org/10.1038/s41563-025-02476-4

Collective photon emission and ferroelectric exciton ordering near Mott insulating state in WSe_2/WS_2 heterobilayers

In the format provided by the authors and unedited

Supplementary Information: Collective photon emission and ferroelectric exciton ordering near Mott insulating state in WSe_2/WS_2 heterobilayers

Luka Matej Devenica†,[1,2] Zach Hadjri†,[1] Jan Kumlin†,[3] Daniel Suárez-Forero**,[2] Runtong Li,[1] Klevis Domi,[2] Bosai Lyu,[2] Weijie Li,[1] Ludivine Fausten,[2] Valeria Vento,[2] Nicolas Ubrig,[2] Song Liu,[4,5] James Hone,[4] Kenji Watanabe,[6] Takashi Taniguchi,[7] Thomas Pohl‡,[3] and Ajit Srivastava*[1,2]

[1]Department of Physics, Emory University, 30322 Atlanta, Georgia, USA

[2]Department of Quantum Matter Physics, University of Geneva, Geneva, Switzerland

[3]Institute for Theoretical Physics, TU Wien, Wiedner Hauptstraße 8-10/136, A-1040 Vienna, Austria

[4]Department of Mechanical Engineering, Columbia University, 10027 New York, New York, USA

[5]Institute of Microelectronics, Chinese Academy of Sciences, Beijing 100029, China

[6]Research Center for Electronic and Optical Materials, National Institute for Materials Science, 1-1 Namiki, Tsukuba 305-0044, Japan

[7]International Center for Materials Nanoarchitectonics, National Institute for Materials Science, 1-1 Namiki, Tsukuba 305-0044, Japan

†These authors contributed equally to this work

*Correspondence to: ajit.srivastava@unige.ch

‡Correspondence to: thomas.pohl@itp.tuwien.ac.at

**Correspondence to: daniel.suarezforero@unige.ch

Supplementary Information Contents:

Figure S1: Sample Details.

Figure S2: The ν_e = 1/7 filling state.

Figure S3: Landé g-factor of the H-stacked IX.

Figure S4: IXX and IX_e circular polarization.

Note 1: Determination of $\nu_{max} = 1$ and calibration of exciton filling.

Figure S5: Schematic of the exciton Mott insulator.

Figure S6: IXX lifetime and detection.

Note 2: Non-monotonic emission rate at short delays and high powers.

Figure S7: Comparison of R- and H- stacked position.

Figure S8: Power-dependent decay dynamics of R-stacked WSe_2/WS_2 heterobilayers.

Note 3: Ruling out photodoping as an origin of IX_d.

Figure S9: The effect of electron doping on IX lifetime.

Note 4: Monte-Carlo and Quantum Jump Simulations.

Note 5: Estimating ν_X with a Gaussian beam profile model.

Figure S10: Gaussian excitation beam profile correction to exciton density estimation.

Figure S11: Sketch of Time-resolved Photoluminescence Spectroscopy (TRPLS) setup.

Figure S12: Lifetime and TRPLS comparison.

Figure S13: Doping and power-dependent PL spectra of an additional H-stacked sample.

Figure S14: Power-dependent PL spectra of additional H-stacked sample with CW excitation.

Figure S15: Power-dependent PL lifetime of additional H-stacked sample.

Figure S16: Lifetime dynamics of the additional H-stacked sample.

Figure S17: Density-dependent IX lifetime and energy dynamics of an additional H-stacked sample.

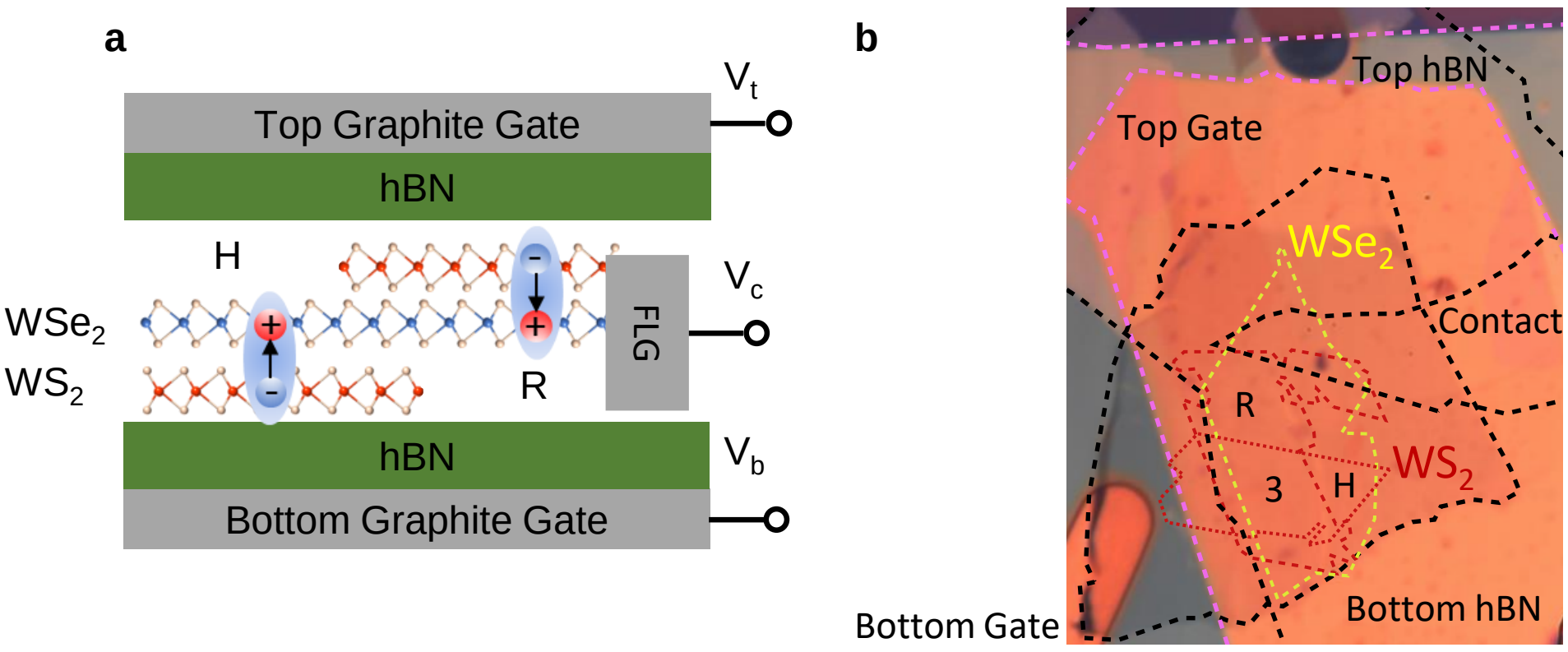

Figure S1: Sample Details. a. Schematic of the primary sample studied. The gating configuration allows for independent control of doping and electrical field in the sample. V_t denotes the top gate voltage, V_b the bottom gate voltage and V_c the direct contact voltage on the sample, which is in our case always kept grounded. The contact voltage is applied to the sample by a few-layer-graphene (FLG) flake. **b.** Optical microscope image of the sample, with regions of different heterobilayer stacking angle indicated as R for R-stacking, H for H-stacking and 3 for the trilayer region, in which the H- and R-stacked outer WS_2 layers overlap. The two regions of this sample are referred to as H-stacked and R-stacked Sample 1 in the main text and supplement. All main text data was collected in this sample.

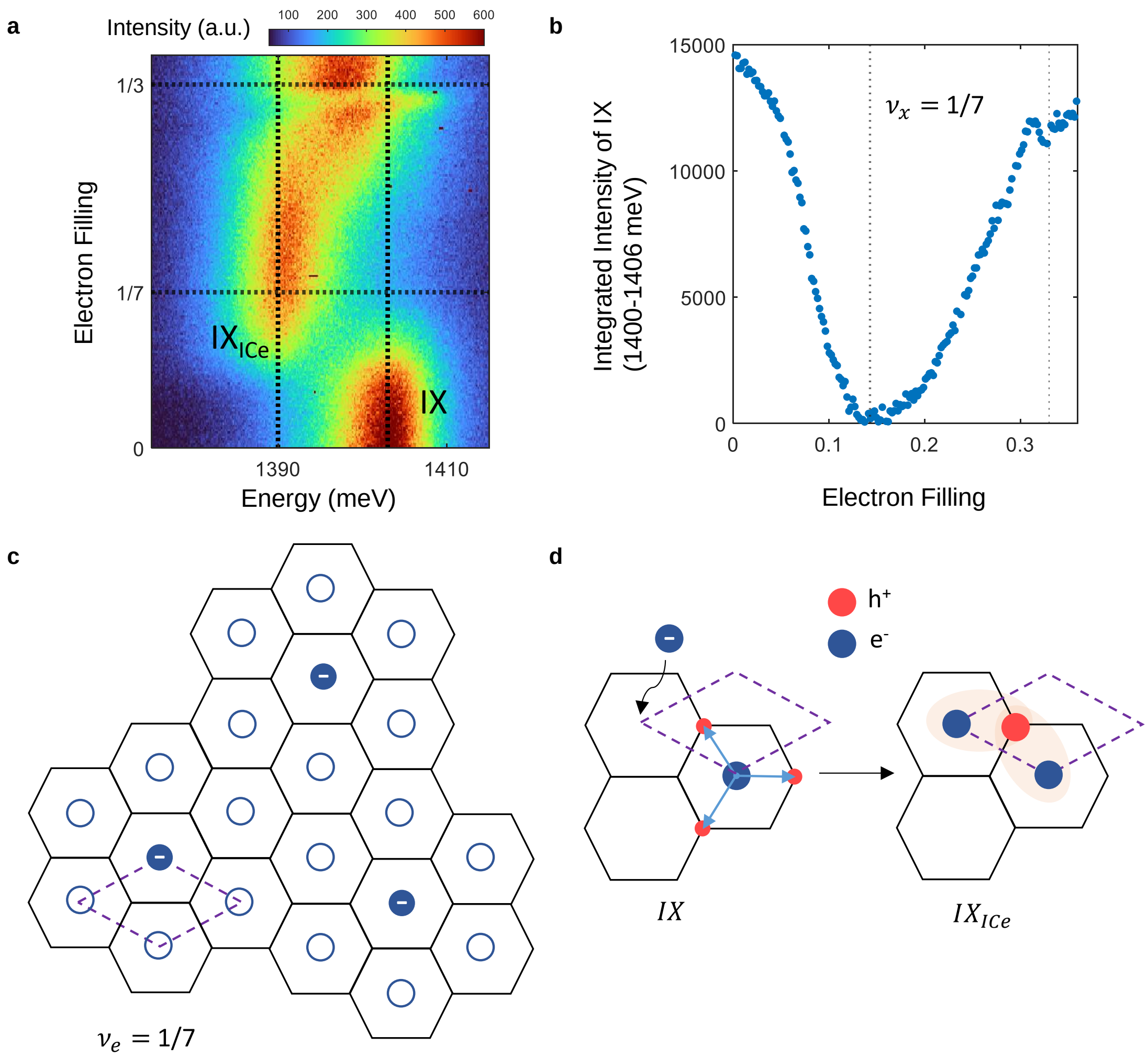

Figure S2: The ν_e = 1/7 filling state. a. Detail of doping-dependent photoluminescence from Fig. 1b of the main text, emphasizing the transition at ν_e = 1/7 doping to the 13 meV red-shifted IX_{ICe}. **b.** Integrated intensity of the IX (non-redshifted) intensity. At 1/7 filling, all IX emission has been red-shifted. **c.** Schematic of 1/7 filling generalized Wigner crystal. A moiré unit cell is indicated in purple dashed lines, and the hexagonal full lines indicate the effective electronic lattice, with each electron site at the center of a hexagon. At 1/7 filling, each unfilled electron site is adjacent to one doped electron such that an IX optically generated in any unoccupied moiré cell will have a nearest-neighbor doped electron. **d.** Schematic of the formation of the IX$_{\mathrm{ICe}}$, described in the main text. The addition of an IX next to a doped electron leads to the formation of an intercell electron-exciton complex. Data from H-stacked Sample 1 measured at 8K.

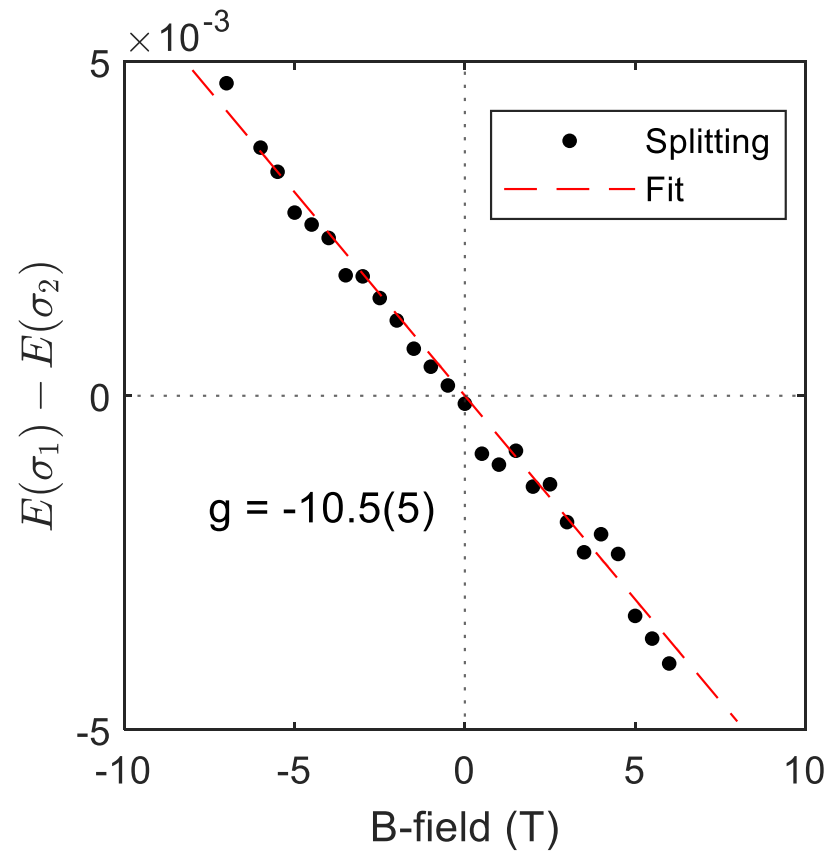

Figure S3: Landé g-factor of the H-stacked IX. a. IX PL peak splitting as a function of magnetic field in H-stacked Sample 1. Magnetic-field dependent splitting of the IX reveals a g factor with a magnitude of 10.5(5). Previous works established the R-stacked g-factor value as close to the monolayer value of 4 [1], and the H-stacked value to be in the 10-14 range [2]. Data from H-stacked Sample 1 measured at 3.8K.

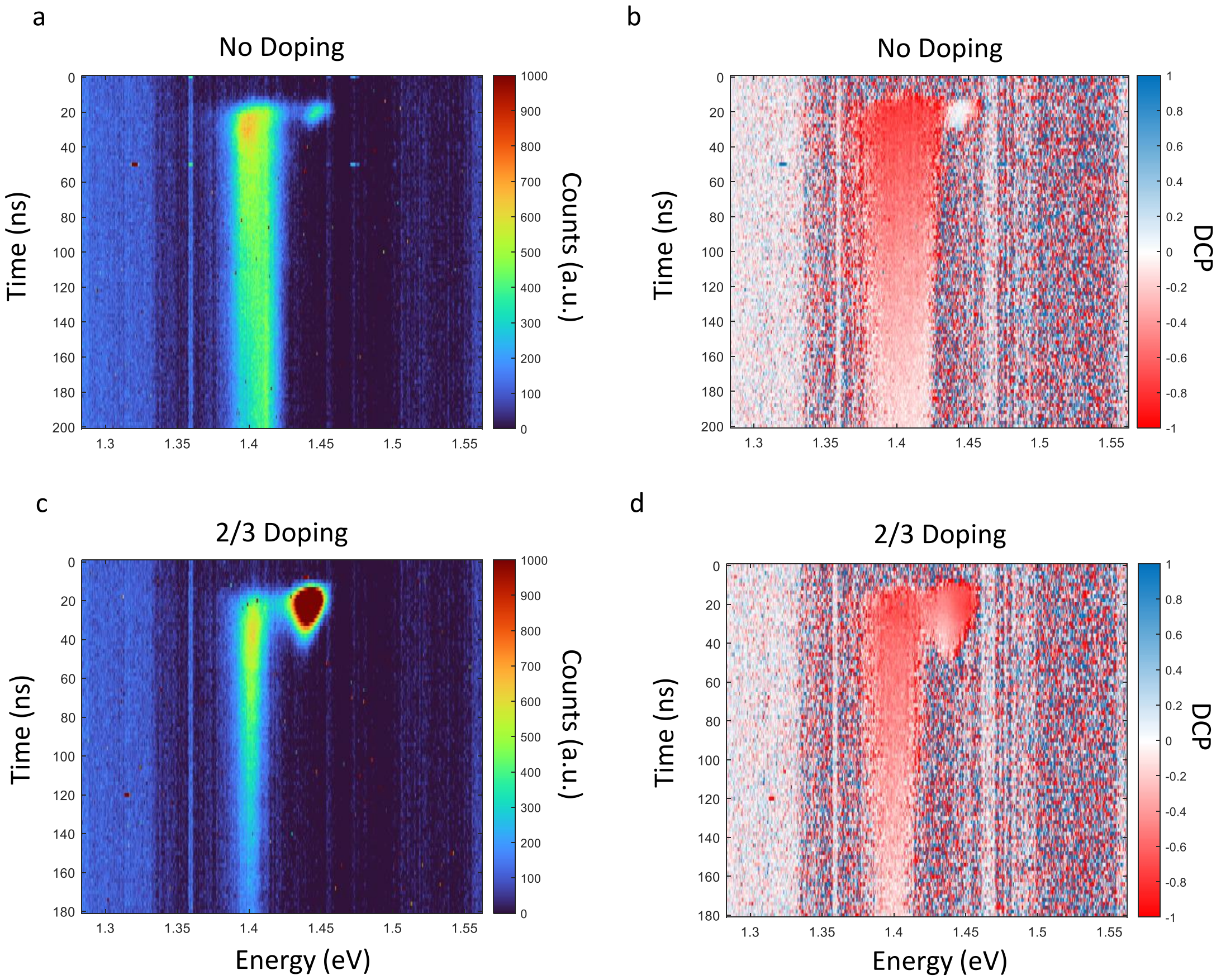

Figure S4: IXX and IX_e circular polarization. **a.** Time-resolved photoluminescence spectra (TRPLS) of the H-position with no doping, with 16 uW of σ^+ polarized excitation power. **b.** Degree of circular polarization (DCP = $\frac{\sigma^+ - \sigma^-}{\sigma^+ + \sigma^-}$) of the emission in panel a. IXX shows a characteristic absence of DCP as expected from the low-energy singlet configuration of the two excitons occupying the moiré site. **c.** TRPLS of the H-position with 2/3 electron filling, with 16 uW of σ^+ polarized excitation power. **d.** Degree of circular polarization (DCP) of the emission in panel c. The high-energy emission consists primarily of IX_e and shows a high degree of DCP. Data from H-stacked Sample 1 measured at 8K.

Supplementary Note 1: Determination of $\nu_{\max} = 1$ and calibration of exciton filling.

In order to analyze our findings, a key parameter is the determination of exciton density in the system. The most readily accessible experimental parameter in this determination is the excitation power at which IXX first appears. In order to relate the density to this power, let us first consider a simple model of our system (Fig. S5). We consider the case of pulsed excitation, when all excitons are generated at once as intralayer excitons and relax quickly ($\sim$ fs) to interlayer excitons (IX). For low excitation power, because of large U_{XX}, the excitons avoid residing in the same moiré site. In other words, empty moiré sites are occupied before double occupancy of any moiré site occurs. This coincides with a linear dependence of emission intensity on excitation power, where the number of generated IX, and therefore their collected intensity, are proportional to the excitation power. However, when all available sites are filled, any additional excitons are forced to an already occupied site, forming IXX. As discussed below, TRPLS allows us to very sensitively detect the IXX peak, even when it is about two orders of magnitude weaker than the IX peak. At this point, IX intensity sharply saturates, as shown in Fig. S5b. A further increase in excitation power simply results in a linear increase of IXX, as is expected from the Mott insulator model shown in Fig. S5a. In our experiment, the Gaussian profile of excitation beam slightly smears out this sharp saturation close to unity filling (see Supplementary Note 5).

Figure 1e of main text, shows this transition from a linear IX regime to saturation, which occurs concurrently with the first appearance of IXX emission. We label the power at which this occurs as corresponding to the density $\nu_{\max} = 1$. As mentioned above, our TRPLS method allows us to achieve a high signal-to-noise ratio in determining when the IXX first begins to appear by measuring the time-resolved spectrum. As shown, Fig. S6a, IXX lifetime is much shorter than that of IX, allowing us to efficiently detect IXX in TRPLS at short times (Supplementary Fig. S6b). Notice that in a time-integrated spectrum, this weak IXX peak will be obscured by the IX tail. Thus, we have high confidence in experimentally determining $\nu_{\max} = 1$.

As shown in Fig. 1d of the main text, the total emitted intensity of the IX excitons below this point of full IX filling is highly linear, which means there is no measurable variation of non-radiative IX losses/exciton diffusion out of the excitation spot, across the range of $0 < \nu < 1$. Therefore, simple linear interpolation is a good method of estimating the initial exciton filling, with a fraction $1/n$ of the excitation power needed to reach $\nu = 1$ leading to an initial filling of

$\nu = 1/n$. Due the fact that our excitation beam is Gaussian, at $\nu_{\rm max} = 1$, the sample region in the center of the beam achieves unit excitonic filling such that IXX forms. However, this may not be case for the regions further away from the beam center. In the linear regime below $\nu_{\rm max} = 1$, we can still safely consider the IX density as depending linearly on the excitation power. This means that the determination of $\rm P_{exc}(\nu_{\rm max} = 1)$ = $11.1(1)$ uW provides a calibration to assign excitonic filling fractions, which becomes proportional to the excitation powers. In other words,

$$\nu_{\rm max} = \frac{P_{\rm exc}}{P_{\rm exc}(\nu_{\rm max} = 1)}.$$

In Fig. 3d of the main text, we report the exciton densities at which the transition from the slow to fast decay occurs in units of $\nu_{\rm max} = 1$.

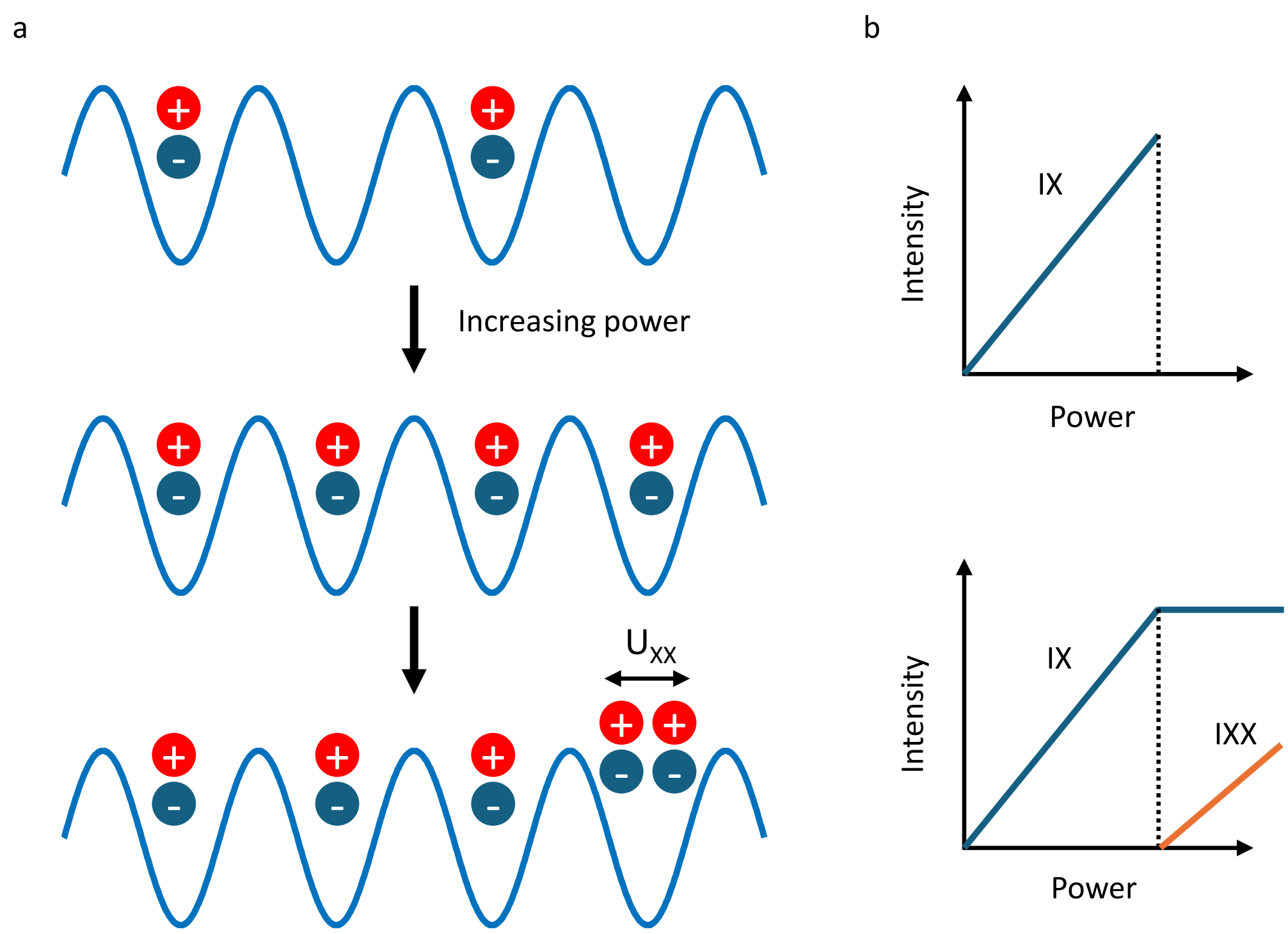

Figure S5: Schematic of the exciton Mott insulator. a. Schematic of sequential filling of excitons in a moiré lattice. The on-site repulsion U_{XX} leads to the excitonic Mott insulator at unit IX filling. **b.** Idealized sketch of an expected intensity profile at different excitation powers before and after saturation.

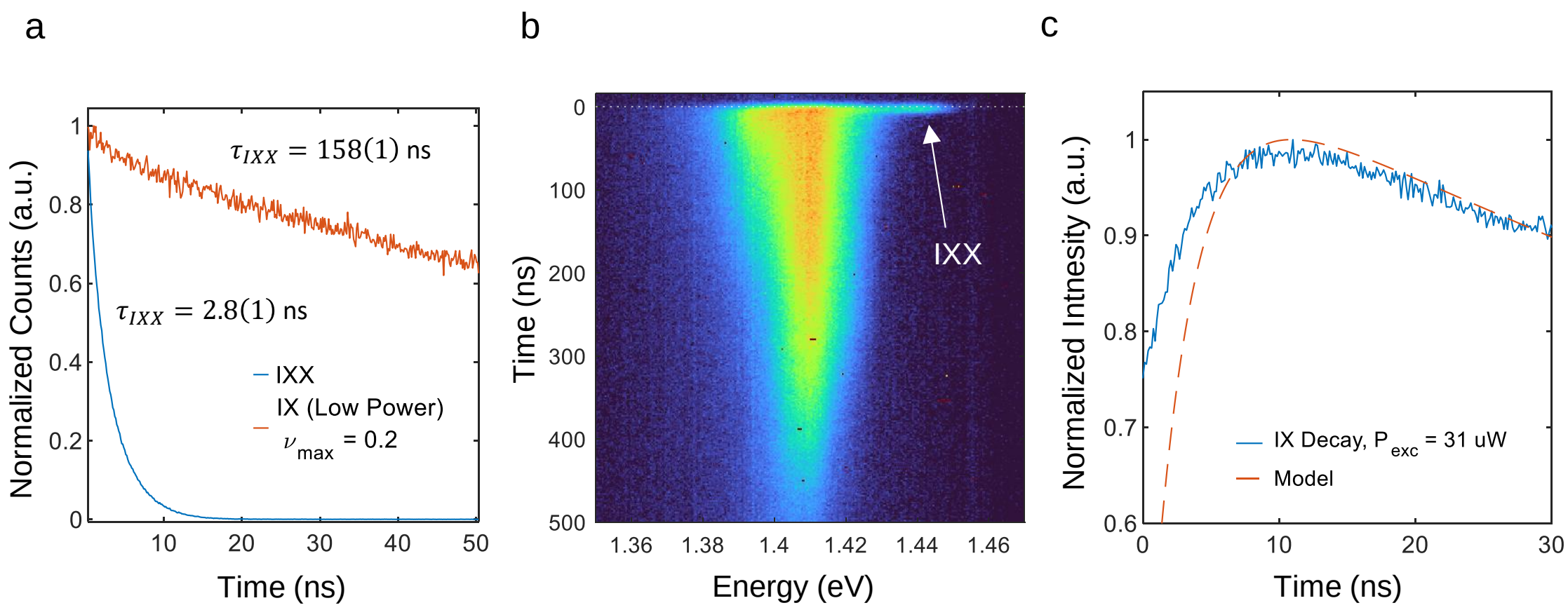

Figure S6: IXX lifetime and detection. a. Comparison of lifetime of IX and IXX. IXX lifetime is about two orders of magnitude shorter. **b.** Time-resolved photoluminescence with 14uW of 640nm excitation. Selecting for the early emission increases the IXX to IX emission ratio, allowing for a precise measurement of when IXX first appears. **c.** Comparison of early (first 30 ns) decay profile of IX at a high excitation power (31 uW) and the IXX 're-pumping' model from Supplementary Note 2. Data from H-stacked Sample 1 measured at 8K.

Supplementary Note 2: Non-monotonic emission rate at short delays and high powers.

For data in Figure 3b of the main text, and particularly the highest power regimes, the substantial presence of IXX at small delays from the excitation laser means that the IX signal is suppressed. This suppression last only briefly, as the IXX sites rapidly decay. In order to fully characterize our lifetime measurements, which only filter for IX emission, we need to account for this effect, which causes a non-monotonic decay profile, with an initial increase in the observed counts before the usual decay. Supplementary Fig. S6c shows the observed counts in the first 30 ns. In order to account for the observed non-monotonic IX emission at early times in high excitation power experiments, we made a simple differential equation model which shows how the rapid decay of the initial IXX population 're-pumps' the IX numbers and leads to the initial 'hump' in our lifetime measurements. The data shown in Supplementary Fig. S6c was taken at the highest measured power, where we can approximate the initial population to be fully IXX across the whole excitation spot. Then, we can initialize the populations of IX and IXX as:

$$N_{\mathrm{IX}}(t=0)=0; N_{\mathrm{IXX}}(t=0)=10000;$$

where 10000 is an approximate number of sites in our excitation spot. The sum $N_{\mathrm{IX}}(t=0)+N_{\mathrm{IXX}}(t=0)$ is constrained by the total number of moiré sites in the excitation spot, which is ~ 1 μm in diameter, and covers approximately 10000 moiré sites (assuming a moiré lattice constant of 8nm). This constraint leads to the observed saturation of the N_{IX}.)

Since we know the radiative rates of IX and IXX (Supplementary Fig. S6a), γ_{IX} and γ_{IXX}, we can employ related rates equations, solving for N_{IX} and N_{IXX} as functions of time:

$$\frac{dN_{\mathrm{IXX}}}{dt}=-\gamma_{\mathrm{IXX}}N_{\mathrm{IXX}}$$
$$\frac{dN_{\mathrm{IX}}}{dt}=\gamma_{\mathrm{IXX}}N_{\mathrm{IXX}}-\gamma_{\mathrm{IX}}N_{\mathrm{IX}}$$

We numerically evaluate the result with a time step much smaller than the lifetimes ($dt=0.01$ ns). In Supplementary Fig. S6c, we compare the calculated intensity of IX emission as a function of time ($I_{\mathrm{calc}}=-N_{\mathrm{IX}}(t)\gamma_{\mathrm{IX}}$) to the observed emission data and find good agreement. It is important to clarify that this model does not account for the saturation of N_{IX}, since we are not including the effects of the moiré lattice. The finite number of moiré sites under the illumination

spot has to be taken into account as a constraint in the maximum values of $N_{\mathrm{IX}}(t = 0)$ and $N_{\mathrm{IXX}}(t = 0)$.

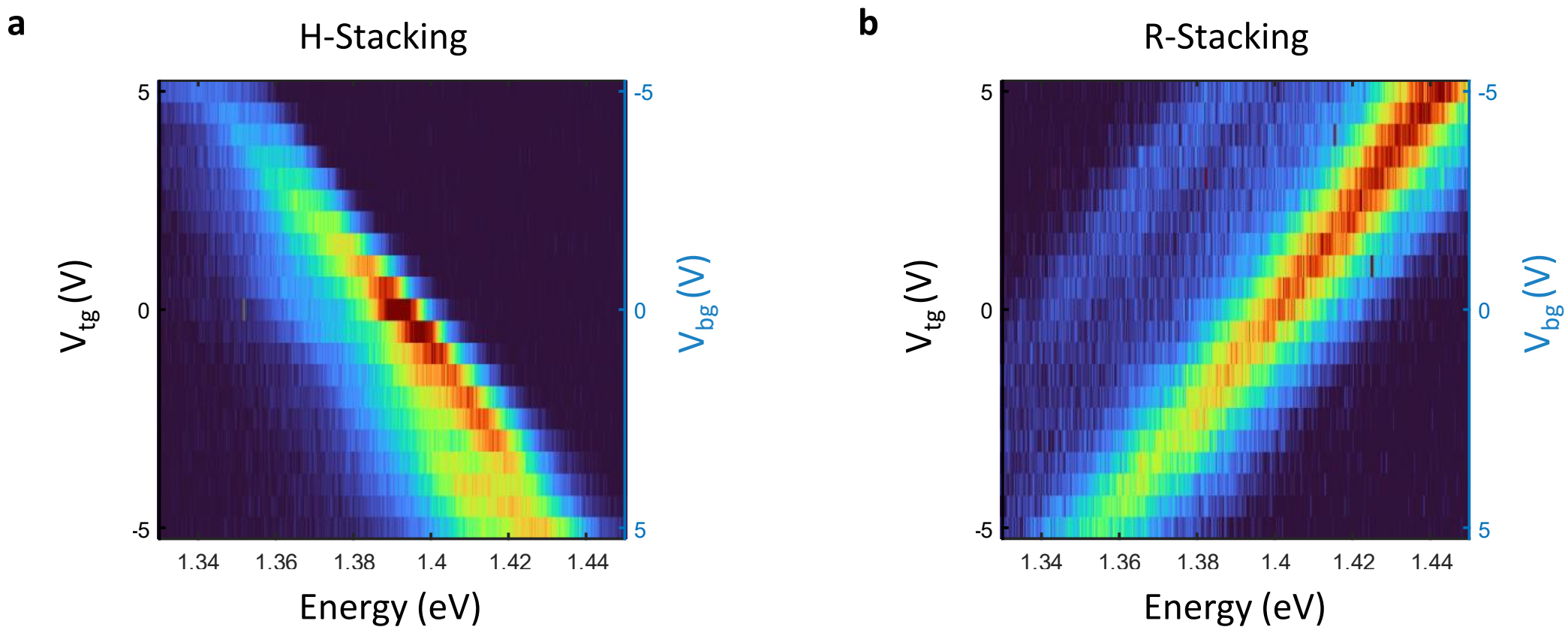

Figure S7: Comparison of R- and H- stacked position. **a,** Electric-field dependence of photoluminescence at the H-stacked position. **b,** Electric-field dependence of photoluminescence at the R-stacked position, showing the opposite dipole to the H-stacked position. Data from H-stacked/R-stacked Sample 1 measured at 8K.

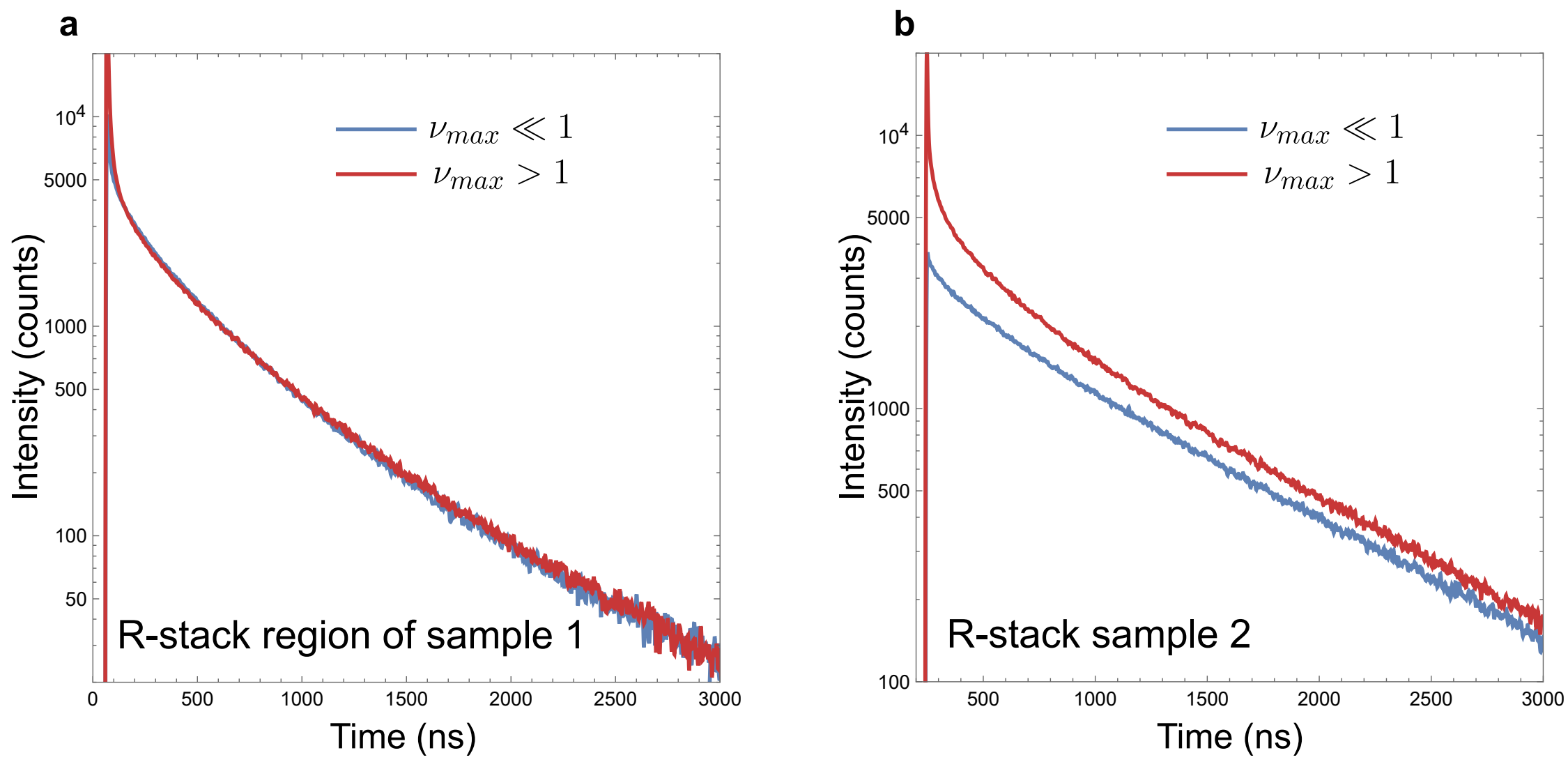

Figure S8: Power-dependent decay dynamics of R-stacked WSe_2/WS_2 heterobilayers. The R-stacked region in Sample 1 (**a**) and another R-stacked Sample 2 (**b**) do not show any observable slow down of decay dynamics with increasing power from 300 nW (170 nW) in **a** (**b**) to the highest available power of $\sim 4\ \mu$W at 200 kHz. Data from R-stacked Samples 1 and 2 measured at 3.8K.

Supplementary Note 3: Ruling out photodoping as an origin of $\mathrm{IX_d}$.

As doping the sample can also lead to a red-shifted emission peak, it is important to rule out the origin of $\mathrm{IX_d}$ from unintentional photodoping. Therefore, we compare the decay lifetime profile observed at high powers (at zero gate voltage) to those at electron dopings at $\nu_\mathrm{e} = 1/6$ (slightly above 1/7). As seen from Supplementary Fig. S9, unlike in the case of $\mathrm{IX_d}$, there is no slowdown observed in the doped case. Thus, we can conclude that the origin of $\mathrm{IX_d}$ is not from unintentional photodoping.

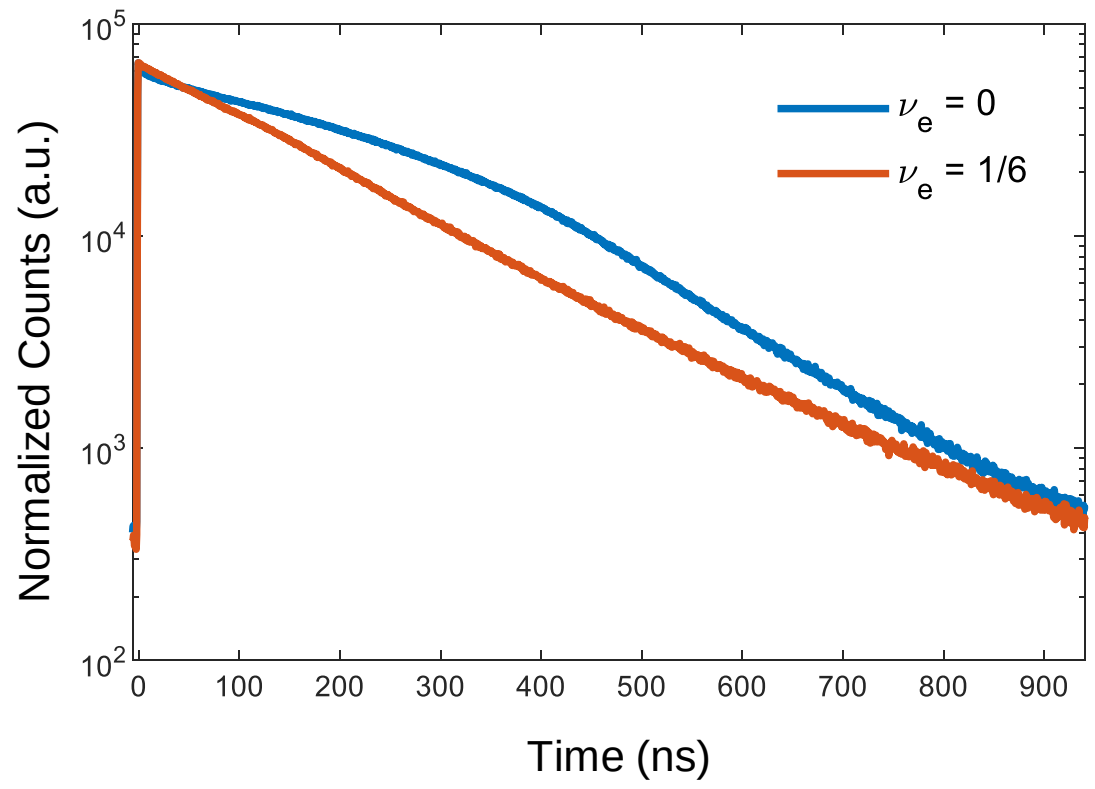

Figure S9: The effect of electron doping on IX lifetime. Comparison of undoped and 1/6 electron-doped exciton lifetime (14 uW, 1MHz pulsed, 640 nm excitation). Data from H-stacked Sample 1 measured at 8K.

Supplementary Note 4: Monte-Carlo and Quantum Jump Simulations

Model: We model the experimental system by considering a triangular lattice with N lattice sites occupied by M excitons. Due to the strong on-site interaction $\mathrm{U_{XX}}$, each lattice site can be occupied by at most one exciton. On each occupied moiré lattice site, for a fixed position of the electron, the hole can be in three different configurations 1, 2, or 3, as shown in Fig. 4a in the main text. We describe the creation of such an exciton at a position $\mathbf{r}_i$ on the lattice by the operator $c^\dagger_{e,i}c^\dagger_{h_\alpha,i}$, where $c^\dagger_{e,i}$ describes the creation of an electron at position $\mathbf{r}_i$ and $c^\dagger_{h_\alpha,i}$ with $\alpha \in \{1,2,3\}$ describes the creation of the corresponding hole in configuration α. Note that all three hole configurations have the same energy.

If two neighbouring lattice sites are occupied by excitons, certain hole configurations are energetically blue-shifted due to the large overlap of the respective hole wavefunctions and the associated large (repulsive) Coulomb energy, $\mathrm{U_{hh}}$.

We describe the system by the Hamiltonian

$$H = \sum_{\langle i,j\rangle} \sum_{\alpha,\beta\in 1,2,3} V_{ij}^{\alpha\beta} c^\dagger_{h_\alpha,i} c^\dagger_{h_\beta,j} c_{h_\beta,j} c_{h_\alpha,i}\,, \tag{1}$$

where the first sum runs over nearest neighbours and $V_{ij}^{\alpha\beta}$ denotes the interaction energy between hole configuration α on site i and hole configuration β on site j. As this interaction energy is the largest scale in our problem, we take it to be infinite whenever the hole wavefunctions overlap for two configurations α and β and zero if not.

In the following, we restrict our analysis to the subspace of eigenstates of zero energy with respect to this Hamiltonian. These eigenstates can, for a given number M of excitons, be charazterized by a spatial configuration $\mathbf{R}_M = \{\mathbf{r}_1, \cdots, \mathbf{r}_M\}$ of the position of the excitons, as well as a hole configuration $\boldsymbol{\alpha} = \{\alpha_1, \cdots \alpha_M\}$ which has zero energy with respect to the Hamiltonian (1). The states are then given by

$$|\varphi_{\mathbf{R}_M,\boldsymbol{\alpha}}\rangle = \prod_{i=1}^{M} c^\dagger_{e,i} c^\dagger_{h_\alpha,i} |\emptyset\rangle \equiv |\alpha_1,\ldots,\alpha_M\rangle\,, \tag{2}$$

where $|\emptyset\rangle$ is the vacuum state with no excitons present.

For a fixed spatial configuration $\mathbf{R}_M$, we take the state

$$|\Psi_{\mathbf{R}_M}\rangle = \frac{1}{\sqrt{\mathcal{N}_{\mathbf{R}_M}}} \sum_{\boldsymbol{\alpha}} |\varphi_{\mathbf{R}_M,\boldsymbol{\alpha}}\rangle \tag{3}$$

to be the strongly correlated many-body state of the electron-hole system. Here, $\mathcal{N}_{\mathbf{R}_M}$ is a normalization constant and the sum runs over all hole configurations with zero interaction energy. Note that the hole delocalization will give rise to a collective decay rate which can be up to three times γ_d.

In order to do so, we note that, while the sum over hole configurations is coherent, different spatial configurations of excitons do not share any coherence and we write the full many-body density matrix for M excitons as

$$\rho^{(M)} = \sum_{\mathbf{R}_M} |\Psi_{\mathbf{R}_M}\rangle \langle \Psi_{\mathbf{R}_M}| \ . \tag{4}$$

The dissipative dynamics of the system is given by the Lindbladian master equation

$$\partial_t \rho = \gamma_d \sum_i \left(\sigma_i^- \rho \sigma_i^+ - \frac{1}{2} \left\{ \sigma_i^+ \sigma_i^-, \rho \right\} \right), \tag{5}$$

where ρ is the many-body density matrix of the exciton system and $\sigma_i^- = \sum_\alpha c_{h_\alpha,i} c_{e,i}$ describes local photon emission due to electron-hole recombination. Note that while the decay described by Eq. (5) is completely local, the delocalization of holes on a single site opens up the possibility of an enhancement of the emission rate if the hole is fully or partially delocalized.

Since the dynamics is completely incoherent, we can decompose the many-body density matrix ρ into different sectors labeled by the exciton number as

$$\rho = \bigoplus_{M=0}^{N} \rho^{(M)} \, . \tag{6}$$

Monte-Carlo Simulations: In order to simulate the dissipative dynamics of the complicated many-body system, we use a combination of Monte-Carlo sampling to approximate the states Eq. (3) and the quantum jump formalism, which will be outlined in the following.

As there is no coherent evolution in the master equation (5), we can describe our system by a series of exciton emission processes at some random times t_j. At any time t between these emission processes, the system has a fixed number of excitons and we assume that the state of the system is described by the density matrix Eq. (4).

Since our Lindblad master equation does not contain any coherent evolution, we can use a simple scheme to calculate the jump times t_j and the overall dynamics. To this end, assume we

are at some time t in a state $|\Psi_{\mathbf{R}_M}(t)\rangle$. For this state, we calculate the emission rate γ_i for a given site i by

$$\begin{aligned}\gamma_i = \gamma_d \langle\Psi_{\mathbf{R}_M}(t)|\, \sigma_i^+\sigma_i^- \,|\Psi_{\mathbf{R}_M}(t)\rangle &= \frac{\gamma_d}{\mathcal{N}_{\mathbf{R}_M}} \sum_{\boldsymbol{\alpha}} \sum_{\boldsymbol{\beta}} \prod_{j\neq i} \alpha_j\beta_j \langle\alpha_i|\, \sigma_i^+\sigma_i^- \,|\beta_i\rangle \\ &= \frac{\gamma_d}{\mathcal{N}_{\mathbf{R}_M}} \sum_{\boldsymbol{\alpha}} \sum_{\beta_i} \langle\alpha_i|\, \sigma_i^+\sigma_i^- \,|\beta_i\rangle \\ &= \frac{\gamma_d}{\mathcal{N}_{\mathbf{R}_M}} \sum_{\boldsymbol{\alpha}'} \sum_{\alpha_i} \eta(\boldsymbol{\alpha}', \alpha_i)\,, \end{aligned} \tag{7}$$

where $\boldsymbol{\alpha}' = \boldsymbol{\alpha}\backslash\{\alpha_i\}$ and $\eta(\boldsymbol{\alpha}, \alpha_i)$ is unity if the local configuration α_i is allowed given the global configuration $\boldsymbol{\alpha}'$ without including site i. Thus, the second sum can either take the value 3, 2, or 1, depending on the neighboring sites.

Given the local emission rates, we can calculate the total emission rate

$$\Gamma = \sum_i \gamma_i\,, \tag{8}$$

where the sum runs only over the occupied sites (alternatively, we can weigh every term by its electron occupation). The total emission rate Γ determines the jump time $t_j = t + t^*$ when one of the excitons recombines with

$$t^* = -\frac{\ln r}{\Gamma}\,, \tag{9}$$

with $0 \leq r \leq 1$ being a random number. The exciton which recombines is determined according to the probability distribution given by

$$p_i = \frac{\gamma_i}{\Gamma}\,. \tag{10}$$

After the exciton recombination, we assume that the hole delocalization process is very fast such that the new state of the system is again well described by the state $\left|\Psi_{\mathbf{R}'_{M-1}}(t+t^*)\right\rangle$ and Eq. (4), with the new spatial configuration $\mathbf{R}'_{M-1}$, where the exciton that has decayed is removed.

The whole simulation procedure can then be summarized by the following steps:

(1) Given a number of excitons M, randomly chose an initial configuration $\mathbf{R}_M$. Below a filling fraction of $1/3$, we use an additional nearest-neighbor exclusion for the excitons to take into account the effect of the static dipole-dipole interactions. This is also motivated by the fact that the emission dynamics does not change for low filling fractions.

(2a) Initialize all holes in the same, but randomly chosen, configuration, that is $\alpha_i = \alpha$, with $\alpha \in \{1, 2, 3\}$.

(2b) Scramble the hole configurations by randomly selecting one of the occupied sites and bring the hole into another allowed configuration. Repeat this step a sufficient number of times to generate one of the states in Eq. (2). This concludes the initialization step.

(3) Calculate the emission rate, jump time, and chose the exciton that decays according to Eqs. (8), (9), and (10), respectively.

(4) Repeat step (3) for the new spatial configuration $\mathbf{R}'_{M-1}$ with one exciton less until no more excitons are present.

(5) Repeat steps (1)-(4) for many randomly chosen initial spatial configurations and average over the different trajectories.

For the simulations, we use a triangular lattice with $N = 30 \times 30$ sites with periodic boundary conditions and chose the initial number of excitons M according to the filling factor $\nu = M/N$.

In order to compare the emission lifetime simulation to our data, we also need to take into account the IXX-related non-monotonic initial part of the decay profile, which is detailed in Supplementary Note 2. Though the IXX lifetime is $\sim$ 100 times shorter than that of IX, and the effect lasts only for $\sim$ 30 ns, a substantial fraction of IX also decay during this time. Since our simulations do not take this effect into account, when comparing the simulation to data at the highest excitation powers, we consider the IX fraction at the end of the IXX-related 'hump', shown in Supplementary Fig. 6c. Therefore, in Fig. 3g of the main text, the initial densities ν_{X} for the data displayed are, in ascending order: 0.16, 0.54, 0.72 and 1, as estimated by the method in Supplementary Note 5. The initial densities ν_X for the simulated curves displayed are, in ascending order: 0.13, 0.53, 0.7 and 0.83. The model density for the highest power data is reduced in order to compensate for the loss of excitons during the initial period while IXX has not fully decayed.

Calculation of emission energy: In order to calculate the emission spectrum using the formalism above, we calculate the electrostatic energy difference ΔE of the hole configuration $\boldsymbol{\alpha}$ immediately before the jump and the configuration $\boldsymbol{\alpha}'$ right after the jump (where, say, the exciton

at position $\mathbf{r}_i$ has been removed). The energy difference is then simply given by the interaction energy of that site i in the current configuration with its surrounding. Due to the fast decay ($\sim 1/r^3$) of Coulomb interaction, we take only the nearest-neighboring sites into account, such that the energy difference for a given hole configuration $\boldsymbol{\alpha}$ is given by

$$\Delta E^{(\boldsymbol{\alpha})} = -\frac{e^2}{4\pi\varepsilon_0\varepsilon_r}\sum_j \Bigg\{ \frac{1}{|\mathbf{r}_i - \mathbf{r}_j|} + \frac{1}{\left|\left(\mathbf{r}_i + \mathbf{r}^{(h)}_{\alpha_i}\right) - \left(\mathbf{r}_j + \mathbf{r}^{(h)}_{\alpha_j}\right)\right|} - \frac{1}{\left|\mathbf{r}_i - \left(\mathbf{r}_j + \mathbf{r}^{(h)}_{\alpha_j}\right)\right|} - \frac{1}{\left|\left(\mathbf{r}_i + \mathbf{r}^{(h)}_{\alpha_i}\right) - \mathbf{r}_j\right|} \Bigg\}, \tag{11}$$

where the sum runs over all occupied nearest-neighbor sites of site i, $\mathbf{r}_i$ denote the electron position, and $\mathbf{r}^{(h)}_{\alpha_i}$ denote the hole position relative to the electron position given a hole configuration α_i.

Similar to the calculations of the emission rate, we obtain a sequence of emission energies $\Delta E(t_j)$. In the limit of $N_{\mathrm{traj}} \to \infty$, we would get a distribution $p(\Delta E)$ at every time t. To account for the finite number of trajectories, we bin different times to approximate this distribution and calculate the spectra shown in Fig. 4d, e in the main text. We made sure that the binning process does not affect the emission spectrum.

Supplementary Note 5: Estimating ν_X with a Gaussian beam profile model

In order to accurately match the initial conditions of our simulation to our data, we must estimate the average excitonic filling over our collection spot, accounting for the non-uniform intensity profile our excitation beam. We attribute the observed smearing (Fig. 1d) of the ideal saturation behavior (Fig. S5b) primarily to this non-uniform intensity profile. As discussed below, taking a Gaussian profile excitation intensity, we are able to match the smeared-out saturation behavior reasonably well (Fig. S9b) and obtain a better quantitative estimate of the initial exciton density ν_X for our simulations (Supplementary Note 4). We emphasize that the deviation from the filling estimate obtained through $\nu_{\max}$ is significant only when saturation begins at higher powers.

As can be seen in Figure 4e-f of the main text, there is an observable difference in the extent of the early redshift we measure at 31 uW and 14 uW, despite the fact that both of these powers are above $P_{exc}(\nu_{\max} = 1)$ = 11.1(1) uW. This is expected in the context of the observation in Fig. 1d of the main text that our IX intensity does not fully saturate across the collection spot at $P_{exc}(\nu_{\max} = 1)$ due to the Gaussian profile of the excitation beam. Therefore, the observed red shift is also expected to saturate only when the IX density reaches unity filling in the entire collection spot.

For this reason, we model the Gaussian excitation profile, as shown in Supplementary Fig. S9a. We use the $P_{exc}(\nu_{\max} = 1)$ power as the estimate of the power needed to locally achieve full filling only at the beam center, and therefore obtain $\nu_{\max}$ (P_{exc}) by proportionality to this power, as described in Supplementary Note 1. Due to the chromatic abberation of the setup, the excitation spot is larger than the collection spot when using the 640nm excitation laser. The total filling ν_X in the collection spot is then calculated as a 2D integral, discarding the excess power that would go into forming IXX:

$$\nu_X = \int_0^R \max(\nu_{\text{loc}}(x), 1)\; 2\pi r dr,$$

where ν_{loc} is the local filling density, given by the Gaussian, and R is the collection spot cutoff. We assume the collection spot has a sharp cutoff, which leads to a reasonable agreement with the saturation behavior at high excitation powers in our measurements (Supplementary Fig. S9b). Using R as the only free parameter in our model, we fit the Gaussian model to the measured intensity, and find that a value of R that equals 1 standard deviation of the Gaussian spot fits

the data well. This allows for a more precise mapping of the observed IX intensity to the initial conditions of our simulations.

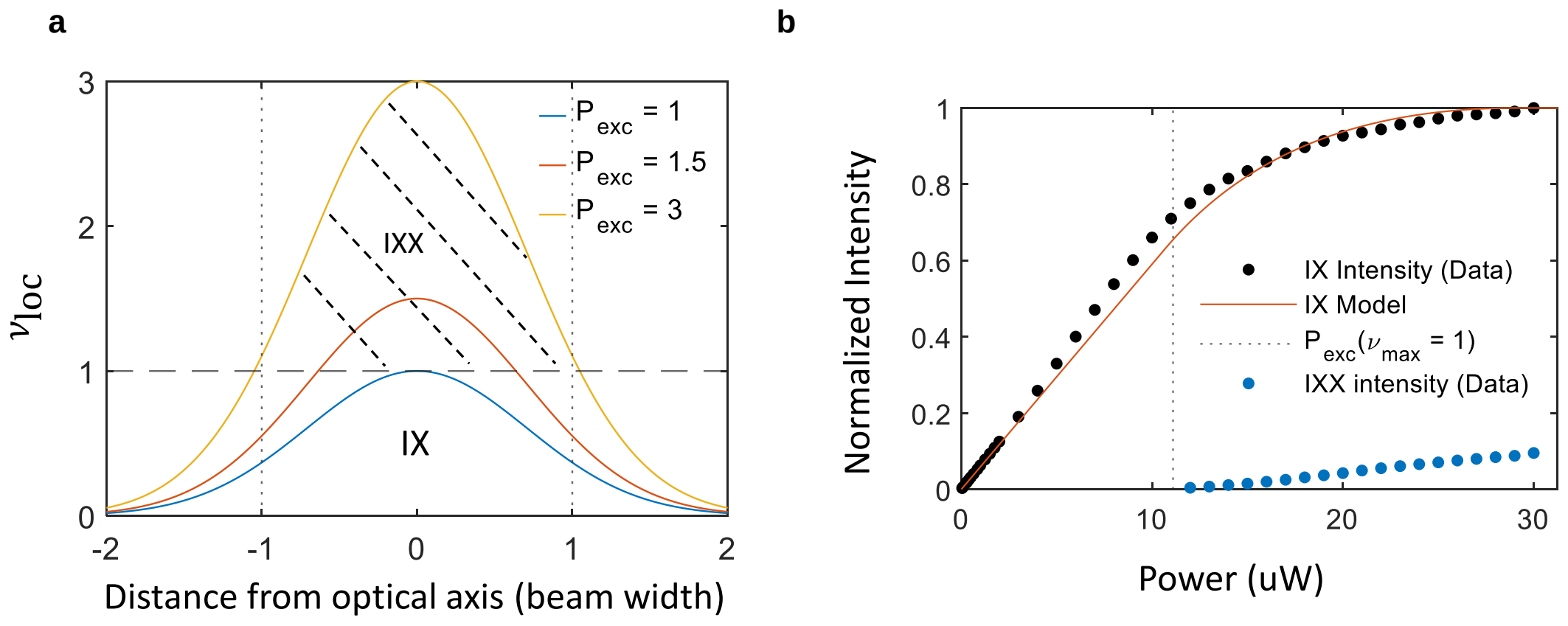

Figure S10: Gaussian excitation beam profile correction to exciton density estimation. a. Model of Gaussian excitation beam leading to nonuniform local excitation density across the excitation spot. The excitation power P_{exc} is expressed in units of $P_{exc}(\nu_{max} = 1)$. **b.** Comparison of measured IX intensity to the model in panel a. Data from H-stacked Sample 1 measured at 8K.

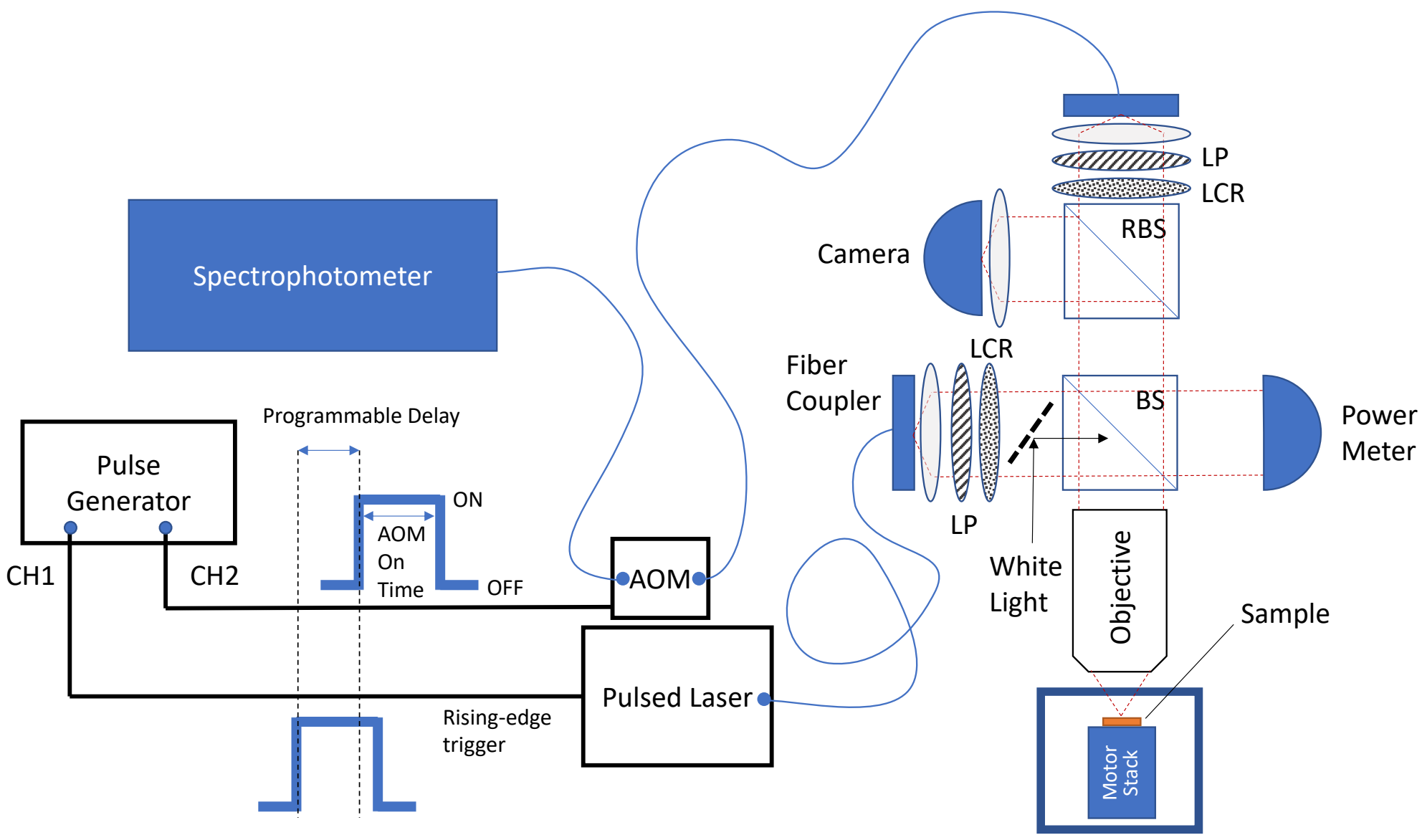

Figure S11: Sketch of Time-resolved Photoluminescence (TRPS) setup. Acronym legend: LCR - liquid crystal retarder; LP - linear polarizer; AOM - acousto-optic modulator; BS - beam-splitter; RBS - removable beamsplitter.

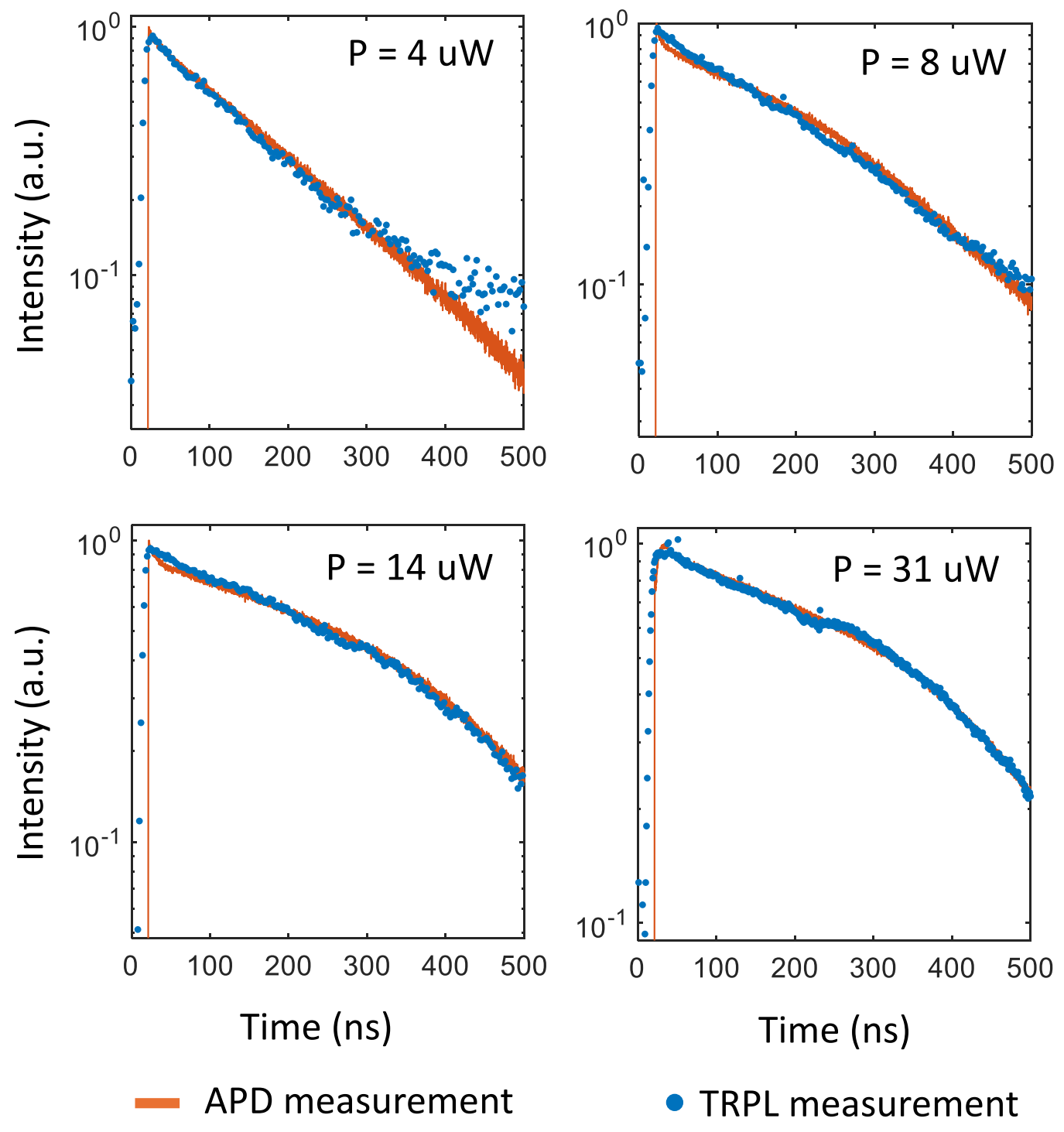

Figure S12: Lifetime and TRPLS comparison. APD-collected lifetime measurements and the integrated intensity of TRPLS with the same filtered emission range overlap well. Intensities are normalized to maximal values. Data from H-stacked Sample 1 measured at 8K.

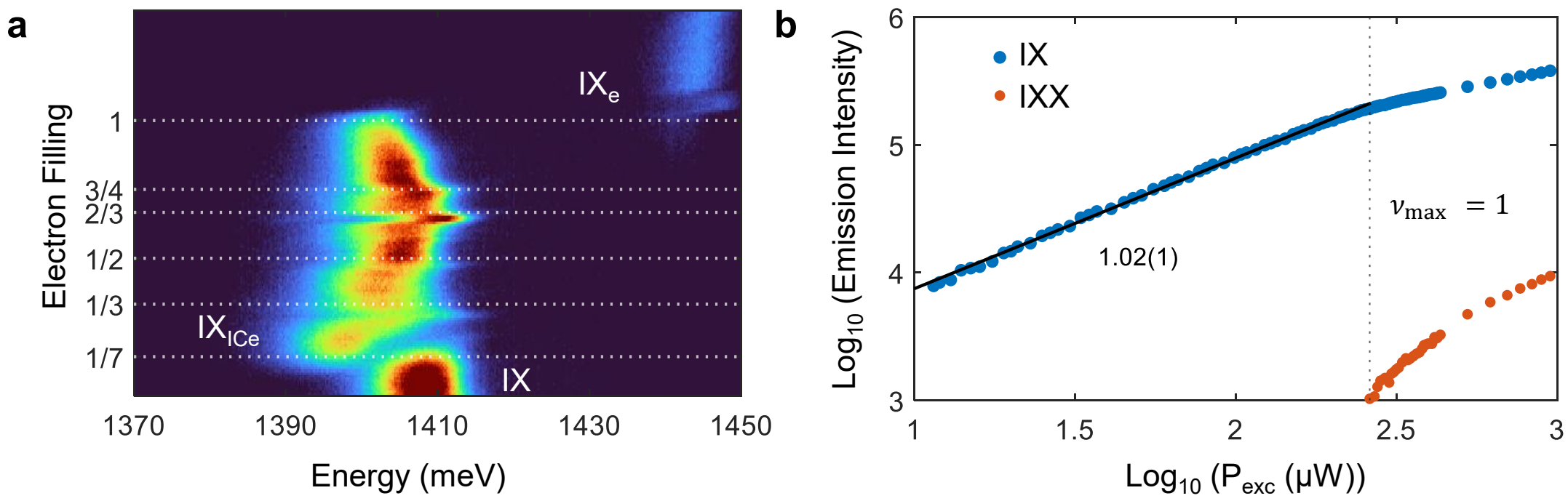

Figure S13: Doping and power-dependent PL spectra of an additional H-stacked sample. a, Doping scan of the photoluminescence (PL) emission of an H-stacked region, using 50 nW of 640 nm 100 kHz laser excitation. Dashed lines indicate generalized Wigner crystals at fractional fillings. **b,** Log-log plot of total intensity of the IX and IXX peaks as a function of excitation power. The intensity of IX increases linearly (slope 1.00 ± 0.01) until the power at which IXX becomes measurable, when IX starts to saturate. Dashed line indicates this point, at which $\nu_{\mathrm{max}} = 1$. Data from H-stacked Sample 2 measured at 3.8K.

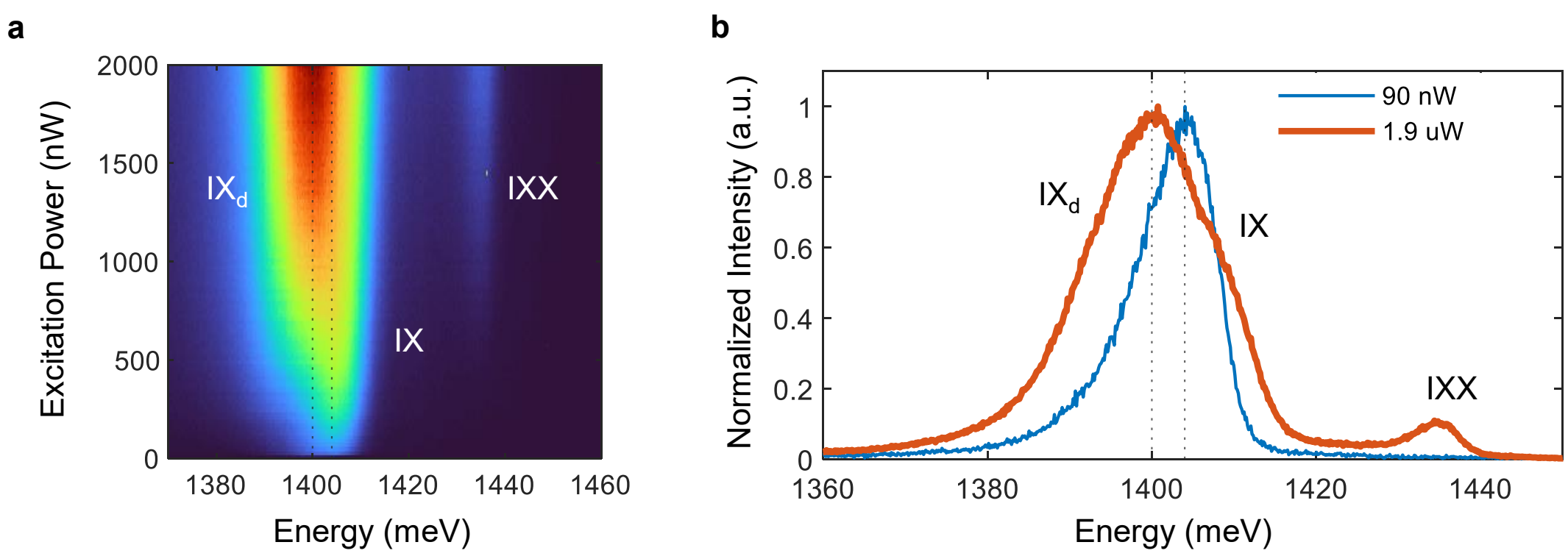

Figure S14: Power-dependent PL spectra of additional H-stacked sample with CW excitation. a, IX PL as a function of excitation (640nm CW laser) power at charge neutrality. Dashed lines indicate IX and IX_d energies at 1404 and 1400 meV, respectively. **b,** Normalized IX PL spectra at a low and high (640nm CW laser) power, in the H-stacked region of the sample. Dashed lines indicate IX and IX_d energies at 1404 and 1400 meV, respectively. Data from H-stacked Sample 2 measured at 3.8K.

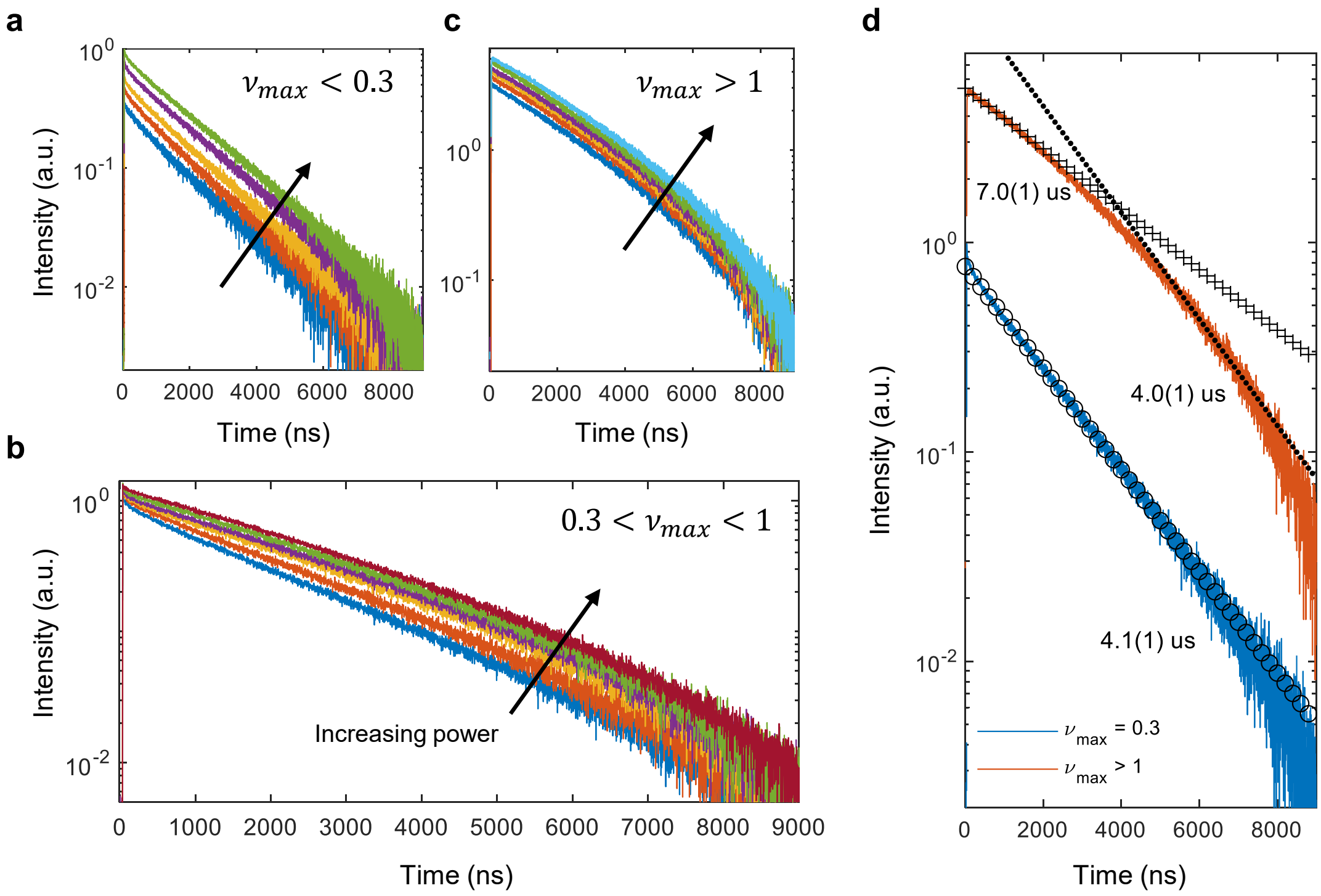

Figure S15: Power-dependent PL lifetime of additional H-stacked sample. a, IX emission decay for the low excitation power regime ($\nu_{\mathrm{max}} < 0.3$). The decay is exponential and lifetime is constant over the entire power range. **b,** IX emission decay for the intermediate excitation power regime (($0.3 < \nu_{\mathrm{max}} < 1$)). The decay becomes initially slower, then returns to the low-density decay rate. **c,** IX emission decay for the high excitation power regime ($\nu_{\mathrm{max}} > 1$). The slowing effect has saturated beyond the excitonic Mott state. **d,** IX emission decay of a high (blue) and low (orange) excitation density case. The fits show the slow and fast decay rates occurring at the high and low density case, respectively. Data from H-stacked Sample 2 measured at 3.8K.

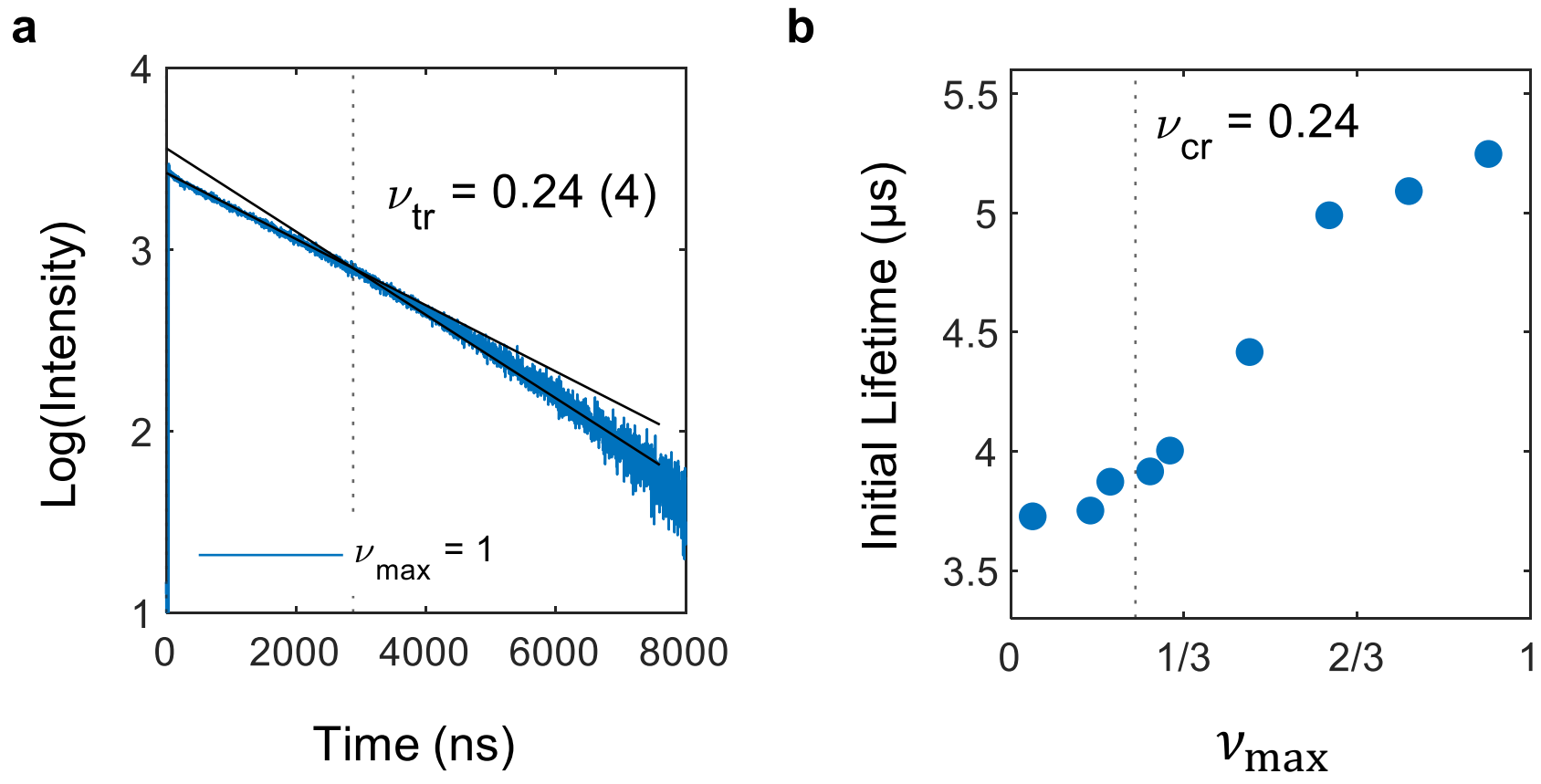

Figure S16: Lifetime dynamics of the additional H-stacked sample. a, IX emission decay of the $\nu_{\rm max} = 1$ case. The fits show the slow and fast decay rates occurring at the high and low density case, respectively. Dashed line indicates the time of transition $t_{\rm tr}$ from the slow to the fast decay regime, corresponding to a density $\nu_{\rm tr} = 0.24(4)$. **b,** Initial (first 1000 ns) lifetime of emission as a function of the initial density $\nu_{\rm max}$. Dashed line indicates the observed $\nu_{\rm tr}$ of 0.24 (4), and coincides with the observation of critical density for changing initial lifetime. Note that $\nu_{\rm tr}$ and $\nu_{\rm cr}$ are expressed in units of $\nu_{\rm max}$. Data from H-stacked Sample 2 measured at 3.8K.

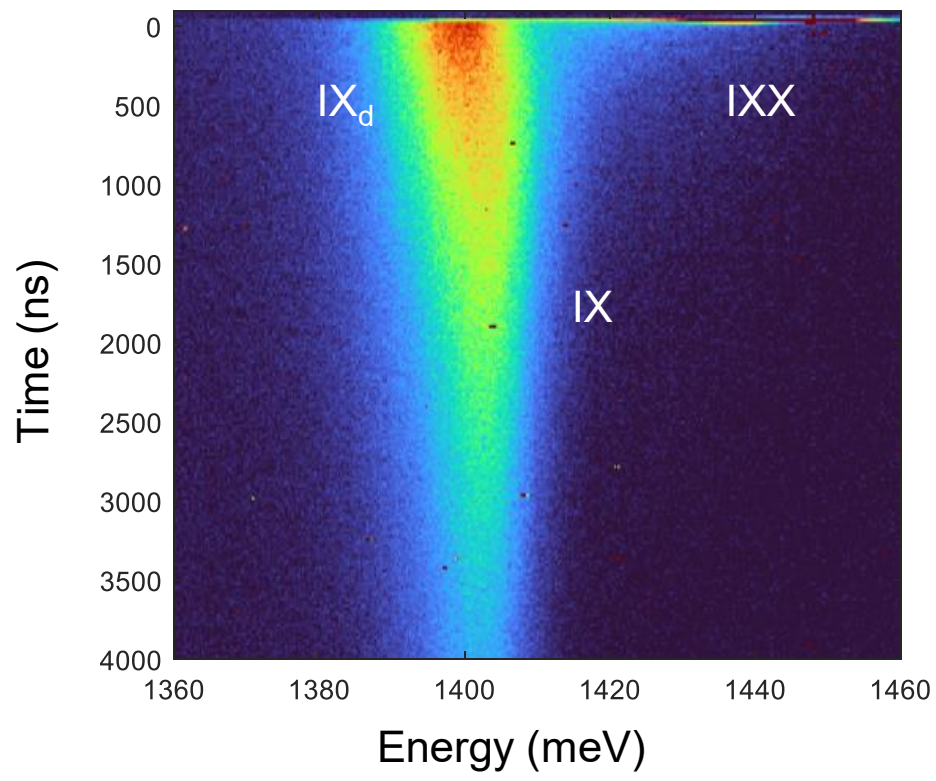

Figure S17: Density-dependent IX lifetime and energy dynamics of an additional H-stacked sample. Time-resolved IX spectrum at $\nu_{\max} > 1$ (excitation power of 4 uW), using a 100 kHz 640nm pulsed laser. Data from H-stacked Sample 2 measured at 3.8K.

---

[1] Tang, Y. *et al.* Simulation of hubbard model physics in wse 2/ws 2 moiré superlattices. *Nature* **579**, 353–358 (2020).

[2] Montblanch, A. R.-P. *et al.* Confinement of long-lived interlayer excitons in ws2/wse2 heterostructures. *Communications Physics* **4**, 119 (2021).
